# Risk Prediction of Three Different Subtypes of Highly Pathogenic Avian Influenza Outbreaks in Poultry Farms: Based on Spatial Characteristics of Infected Premises in South Korea

**DOI:** 10.3389/fvets.2022.897763

**Published:** 2022-05-31

**Authors:** Dae-sung Yoo, Byung Chul Chun, Kwan Hong, Jeehyun Kim

**Affiliations:** ^1^Department of Animal Disease Control and Quarantine, Graduate School of Public Health, Korea University, Seoul, South Korea; ^2^Division of Veterinary Epidemiology, Animal and Plant Quarantine Agency, Gimcheon, South Korea; ^3^Department of Preventive Medicine, College of Medicine, Korea University, Seoul, South Korea; ^4^Transdisciplinary Major in Learning Health Systems, Department of Healthcare Sciences, Graduate School, Korea University, Seoul, South Korea

**Keywords:** avian influenza, highly pathogenic avian influenza, HPAI, machine learning, risk assessment, spatial analyses

## Abstract

From 2003 to 2017, highly pathogenic avian influenza (HPAI) epidemics, particularly H5N1, H5N8, and H5N6 infections in poultry farms, increased in South Korea. More recently, these subtypes of HPAI virus resurged and spread nationwide, heavily impacting the entire poultry production and supply system. Most outbreaks in poultry holdings were concentrated in the southwestern part of the country, accounting for 58.3% of the total occurrences. This geographically persistent occurrence demanded the investigation of spatial risk factors related to the HPAI outbreak and the prediction of the risk of emerging HPAI outbreaks. Therefore, we investigated 12 spatial variables for the three subtypes of HPAI virus-infected premises [(IPs), 88 H5N1, 339 H5N8, and 335 H5N6 IPs]. Then, two prediction models using statistical and machine learning algorithm approaches were built from a case-control study on HPAI H5N8 epidemic, the most prolonged outbreak, in 339 IPs and 626 non-IPs. Finally, we predicted the risk of HPAI H5N1 and H5N6 occurrence at poultry farms using a Bayesian logistic regression and machine learning algorithm model [extreme gradient boosting (XGBoost) model] built on the case-control study. Several spatial variables showed similar distribution between two subtypes of IPs, although there were distinct heterogeneous distributions of spatial variables among the three IP subtypes. The case-control study indicated that the density of domestic duck farms and the minimum distance to live bird markets were leading risk factors for HPAI outbreaks. The two prediction models showed high predictive performance for H5N1 and H5N6 occurrences [an area under the curve (AUC) of receiver operating characteristic of Bayesian model > 0.82 and XGBoost model > 0.97]. This finding emphasizes that spatial characteristics of the poultry farm play a vital role in the occurrence and forecast of HPAI outbreaks. Therefore, this finding is expected to contributing to developing prevention and control strategies.

## Introduction

The avian influenza virus, a member of the Orthomyxoviridae family, is primarily classified as highly pathogenic avian influenza virus (HPAIv) and low pathogenic avian influenza virus (LPAIv) based on the degree of pathogenicity in chickens according to the World Animal Health Organization (OIE). Chickens or turkeys exposed to HPAI develop acute systemic symptoms that lead to death (1). The virus has been considered a zoonotic pathogen with a high potential to develop into a pandemic disease for humans, resulting in numerous casualties and massive economic instability. According to the World Health Organization, the number of confirmed H5N1 and H7N9 cases in humans is 861, with 455 deaths, and 1,568, with 616 deaths, respectively, as of April 3, 2020. In addition to the overall fatality rates of avian influenza, the human case fatality rate of HPAIv has been reported to be significantly higher than LPAIv (2).

The main route of transmission of HPAIv to humans is direct or indirect contact with live or dead poultry infected with the virus in live bird markets (LBMs), backyard poultry flocks, or direct contact with fluids that contain the virus (1, 3, 4). A recent epidemiological study suggested that the proximity of a domestic poultry cage or nesting area to the house and domestic poultry transportation to markets are significant risk factors for H5N1 infection in humans, Cambodia (5). Thus, the fundamental approach to mitigating human infections and preventing this disease from mutating into a novel type of pathogen that becomes a pandemic is to control viral circulation at poultry production sites.

Since the first case was reported at a domestic chicken farm on December 10, 2003, South Korea has experienced multiple HPAI epidemics that resulted in more than 1,100 cases with the three different subtypes of HPAIv: H5N1, H5N8, and H5N6. From 2008 to 2011, a single subtype (H5N1) of < 100 cases were confirmed in poultry holdings across the nation within 140 days of each epidemic (6). However, since 2014, HPAI epidemics have tended to be prolonged and have heavily affected poultry operations across the country. An economic report maintained that direct and indirect financial losses were estimated to account for approximately 295 million US dollars during the 2014-15 HPAI epidemic (7). In particular, HPAIv H5N6 from November 2016 to March 2017 with the worst epidemic producing 343 cases and causing 36% of layer farms across the country to depopulate (8). Furthermore, during the 2016-17 HPAI H5N6 epidemic, the total incidence reached 343 cases within 106 days, leading to severe economic losses of about 238 million US dollars in direct compensation for poultry depopulation. Approximately 36.0% of chicken layer farms and 51.5% of layer breeding farms were depopulated because of the unprecedented massive outbreaks during the 2016-17 HPAI H5N6 epidemic, resulting in price instability and a loss of food security (8).

Interestingly, the spatial distribution of HPAI outbreaks in Korea appears to be concentrated in the small southwestern region, Naju, and its central region, Eumseong, where the majority of domestic ducks in Korea are produced irrespective of the subtype of the virus (6, 9, 10). Moreover, phylogenetic studies on HPAIv have indicated that domestic duck farms are highly susceptible to the first infection by HPAIv and amplify the virus for lateral transmission among all poultry species farms in South Korea (9, 10). In this context, it is arguable that geographical factors, including domestic duck farm density, are strongly linked to the HPAI epidemics in Korea. Numerous studies have found that spatial factors significantly increase the risk of HPAI infections during poultry operations in Asian regions. For example, in China, the density of domestic waterfowl, proportion of land occupied by water, and density of the human population are significant risk factors for HPAIv H5N1 infection (11). Additionally, one study reported that LBMs in rural areas are associated with increased H7 subtypes in Zhejiang province (12). Similarly, in Vietnam, larger rice paddy fields around a poultry farm, closer distance between wetlands and poultry farms, and greater density of free-grazing duck farms had a positive association with HPAI infections in poultry farms (13–16). In addition to the argo-environmental characteristics around poultry farms, a decreasing distance to the closest major road increased avian influenza virus prevalence (17).

The poultry production environment in Korea has similar and distinctive characteristics to those of Asian countries, where identical or different spatial factors contribute to the risk of HPAI. For example, a large proportion of land is covered by rice fields (8.44% of the total territory) around poultry farms, and a relatively large proportion of domestic duck farms (16.0% of the total poultry farms). On the other hand, domestic ducks are commonly raised indoor, thus they hardly have direct contact with wild birds. Furthermore, most poultry farms have large and medium-scale production capacities. For example, 97.6% of domestic duck farms and 92.3% of chicken farms produced more than 5,000 and 10,000 birds, respectively for the first quarter of the year 2020, according to livestock survey of the Korea Institute for Animal products quality Evaluation (18).

Additionally, the different subtypes of HPAIv presented heterogeneous pathogenicity to poultry and shedding levels, which is assumed to differently influence the time to report the detection of infection period and, consequently, the magnitude of the epidemic. For example, the H5N8 subtype occurring in 2014–2016 caused relatively mild clinical symptoms in duck species, compared with the H5N6 subtype in the 2016–2017 epidemic (19). In contrast, the H5N1 subtype that affected poultry holdings during the 2010–2011 epidemic was lethal to domestic ducks (20). Furthermore, the basic reproduction number, which estimates the average number of infectees by a single infector, was the highest in H5N1 compared to subtype H5N8 and H5N6 (21).

Hence, at the expected emergence of the new HPAI (22), it is crucial to identify common spatial risk factors associated with different HPAI occurrences and build a risk prediction model for prevention and response strategies specific to Korea. However, none of the studies have investigated the association between spatial characteristics and diverse subtypes of HPAI infections at poultry farms at the national level or conducted risk assessment from a geographical standpoint to provide a scientific basis for prevention strategies. In this regard, there are two objectives for this study: to improve the understanding of HPAI risk factors and provide a scientific basis for implementing a risk-based surveillance system for poultry farm production and ultimately preventing human infection. The first one is to investigate the spatial characteristics of the 2014–2016 H5N8 epidemic, the longest outbreaks affecting the largest number of poultry farms, such as the distance to habitats of migratory birds, size of farmland, the density of poultry farms, and human. Risk prediction models were built based on the significant spatial factors associated with HPAI infections using a case-control study of 339 H5N8-infected and 626 non-infected poultry farms. Lastly, we predicted the risk values in 88 HPAI H5N1 and 335 H5N6 infections using the model built based on spatial risk factors relevant to H5N8 and compared the observed data with the prediction to validate the accuracy of the risk prediction model for different subtypes of HPAI from retrospective and prospective perspective.

## Materials and Methods

### Study Frame

**Figure 1:**
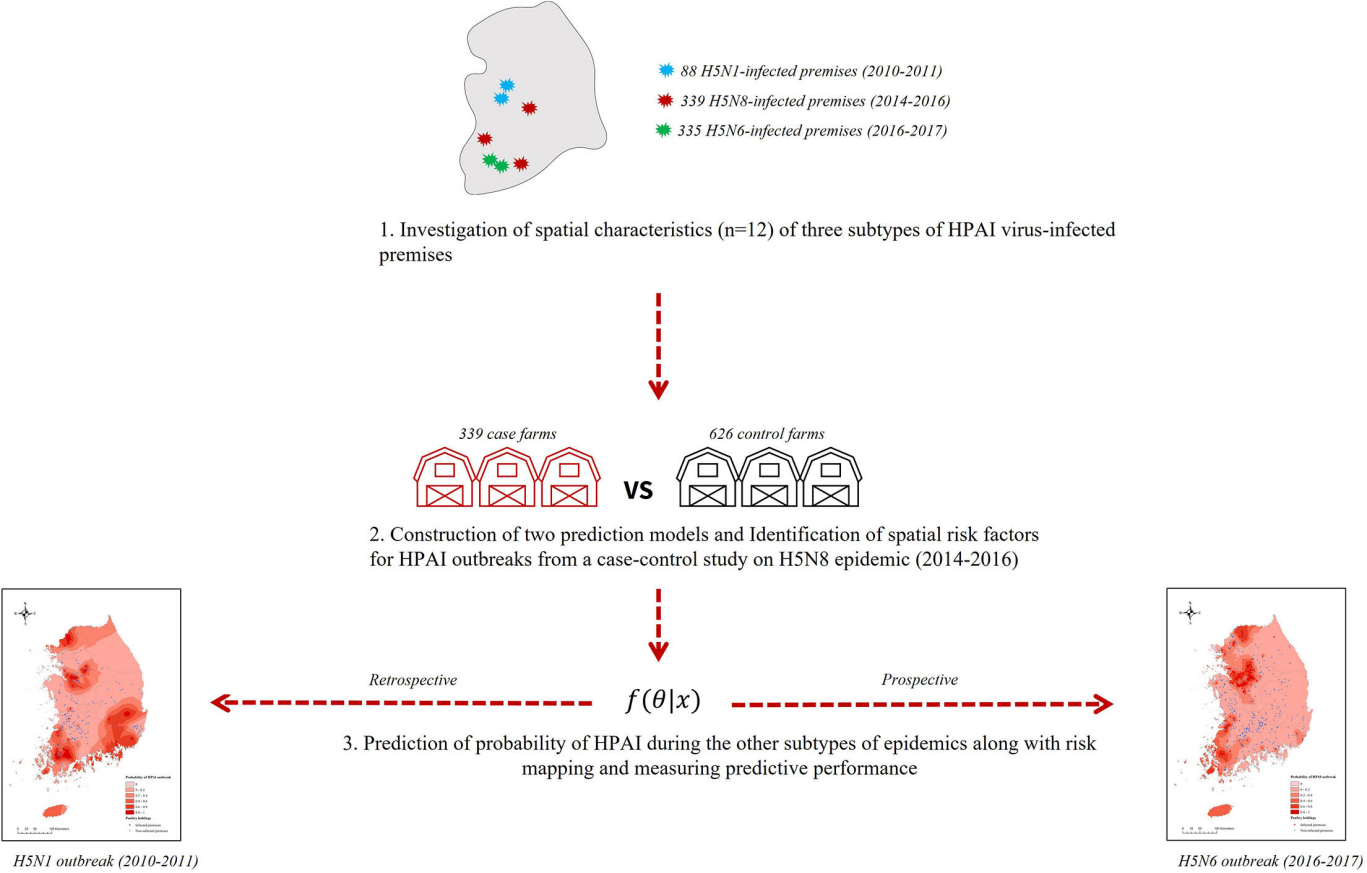
Study frame for the risk prediction of three subtypes of highly pathogenic avian influenza outbreaks in poultry farms.

As illustrated in Figure 1, we first investigated the spatial characteristics of the three subtypes (H5N1, H5N8, and H5N6) of HPAIv IPs during each epidemic in South Korea. Next, the prediction model was built from a case-control study on the HPAI H5N8 epidemic from 2014 to 2016 to identify spatial risk factors associated with HPAI incidence in chicken or domestic duck holdings. Finally, with the significant risk factors, the remaining subtypes of the HPAI outbreaks were predicted using a statistical and machine learning model, followed by creating a risk map across the nation.

### Three HPAI Subtypes Outbreak Data

**Figure 2:**
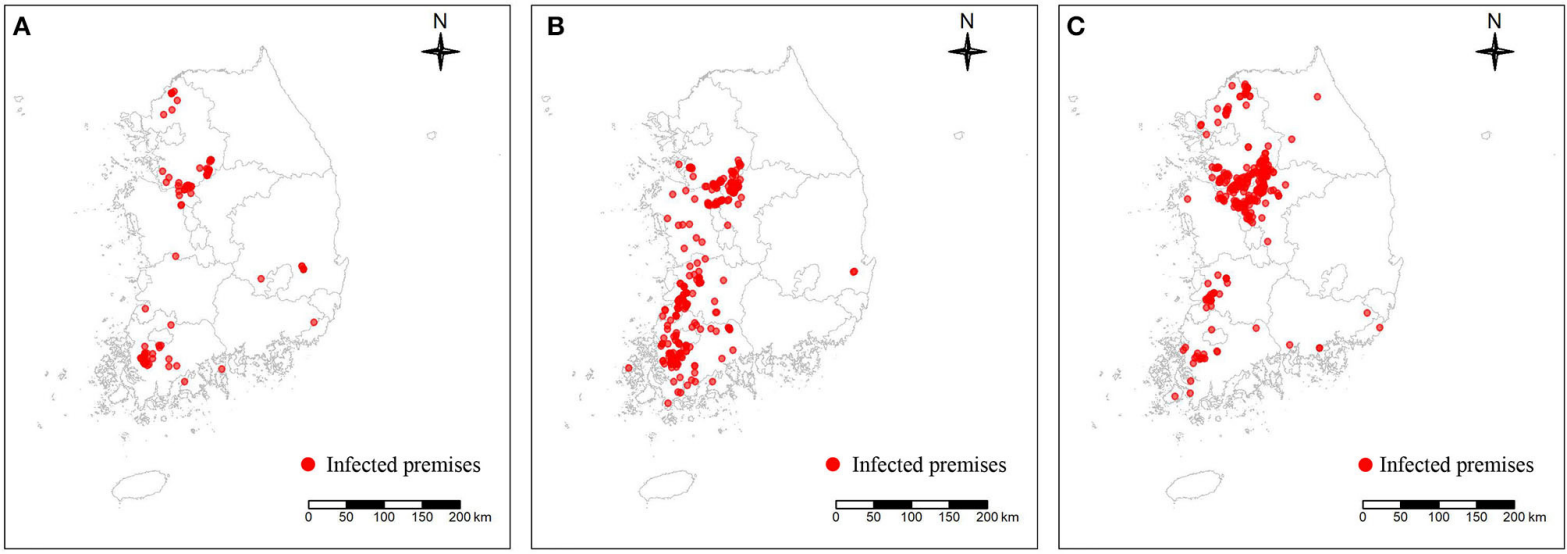
Geographical distribution of three different subtypes of highly pathogenic avian influenza-infected premises denoted by a red dot: H5N1 subtype **(A)**, H5N8 subtype **(B)**, and H5N6 subtype **(C)**.

In this study, three different subtypes (H5N1, H5N8, and H5N6) of the HPAI epidemic in South Korea were included to examine the spatial characteristics of the IPs and predict the risk of occurrence, as summarized in Table 1. As shown in Figure 2, the data on IPs during each epidemic, limited to chicken and domestic duck farm (88 IPs for H5N1, 339 IPs for H5N8, and 335 IPs for H5N6), which consisted of the date of confirmation, geographical coordinates, and farm species, were obtained from the Korea Animal Integrated System (KAHIS) of the Animal and Plant Quarantine Agency, South Korea. On the other hand, the data on non-IPs producing either chicken or domestic duck farms (201 non-IPs for H5N1, 626 non-IPs for H5N8, and 335 non-IPs for H5N6 epidemic) for each epidemic contained geographical coordinates and farm species, compiled from the KAHIS.

**Table 1:** Overview of highly pathogenic avian influenza epidemics targeted for comparative risk assessment in South Korea.

**Epidemic**	**2010/11**	**2014/16**	**2016/17**
Subtype	H5N1	H5N8	H5N6
H5-clade	2.3.2.1	2.3.4.4	2.3.4.4
Duration (unit: days)	139	562	108
No. of infected chicken farms (%) ^†^	34 (37.4%)	93 (23.7%)	197 (57.4%)
No. of infected domestic duck farms (%) ^†^	54 (59.3%)	294 (74.8%)	138 (40.2%)
No. of infected other poultry species farm^‡^	3 (3.3%)	6 (1.5%)	8 (2.3%)
Total no. of cases	91	393	343
No. of poultry culled (unit: birds)	6,473	19,311,634	37,870,000

*The farm species is defined as the major poultry species raised in the infected premises in terms of flock size*.

† *The percentage in the parenthesis was equal to the proportion of given species farms over total cases*.

‡ *Other types of poultry farm consisted of quail, goose, pheasant, and other indigenous species*.

### Spatial Variable Data

The spatial variables associated with HPAI occurrence on a poultry farm were selected from previous research on risk factors (12–14, 23–27) and epidemiological survey reports issued by the Ministry of Agriculture, Food and Rural Affairs in South Korea (28–30). As a result, 12 variables were chosen to examine the spatial characteristics of the HPAI outbreaks in farms (Table 2). All spatial variables were measured using continuous scale values. The spatial resolution and time comparability of the data was central to improving the validity of the analyses. Therefore, we derived the spatial variables from the various data sources that provided fine geographical resolution and matched the time period to each of the HPAI epidemics (2010–2011 for H5N1, 2014–2015 for H5N8, 2016–17 for H5N6) to accommodate the landscape change, as noted in Table 2.

**Table 2:** List of spatial variables used for risk prediction of highly pathogenic avian influenza outbreak in poultry farms in South Korea.

**Variable (*n* = 12)**	**Unit**	**Data source (Period)**	**Note**
Topology			
Elevation	m	USGS SRTM (2014)	All subtype
Topological wetness index^†^			All subtype
Land-use/cover			
Proportion of forest within a 3 km radius	%	Ministry of Environment, South Korea (2009, 2017)	2009 data for H5N1, 2017 data for H5N8, H5N6
Proportion of rice field within a 3 km radius	%		
Proportion of waterbody within a 3 km radius	%		
Proportion of wetland within a 3 km radius	%		
Minimum distance to driveway	km	National Geographic Information Institute, South Korea (2010, 2014, 2016)	2010 data for H5N1, 2014 data for H5N8, 2016 data for H5N6
Poultry and human			
Human	No. of inhabitant/10 km^2^	Worldpop (2011, 2014, 2016)	2011 data for H5N1, 2014 data for H5N8, 2016 data for H5N6
Chicken farm	No. of farms/km^2^	Korea Animal Health Integration System (2016)	
Domestic duck farm	No. of farms/km^2^		All subtype
Wildlife and live bird market			
Minimum distance to major migratory birds‘ habitats for wintering	km	Ministry of Environment, South Korea (2014)	All subtype
Minimum distance to live bird market	100 m	Ministry Agriculture, Food and Rural affairs, South Korea (2014)	All subtype

*USGS, United States Geographical Survey; SRTM, Shuttle Radar Topography Mission*.

† *Topological wetness index indicates the level of water accumulation and local drainage as a function of the total catchment area, flow width and slope gradient*.

The elevation of poultry holdings in the study was derived from the digital elevation model (DEM) data obtained from the Shuttle Radar Topography Mission of the United States Geological Survey in 2014. From the DEM data, the topological wetness index (TWI) across the nation was estimated, and the value of individual poultry holdings was extracted based on its geographical coordinates. The TWI index is an indicator of water accumulation and local drainage, defined as a function of the total catchment area, flow width and slope gradient [calculated as ln(α/tanß), where α is the total catchment area (i.e., a cumulative upslope area that supplies water) per-flow width, and tanß is the surface slope gradient between the cell in upstream and a cell further downslope]. It is often used as a proxy for soil moisture because it integrates the water supply from upslope and downslope water drainage. The proportion of the forest, rice paddy fields, water bodies, and wetlands within a 3km-radius from each study farm were calculated using the national land cover map at the 5 m spatial resolution provided by the Environmental Spatial Information Service, Ministry of Environment, South Korea, in 2009 and 2017. The determination of the radius range (3 km) for the proportion of each type of land cover was made according to the standard operative procedure for HPAI, South Korea, where the area within a 3-km radius from an HPAI-infected premise or localities of HPAI-positive surveillance area in wild birds was classified as a biosecurity zone mainly due to the high risk of local spread. Additionally, data on driveways were retrieved from the Korea National Transport Information Center in 2010, 2014, and 2016. Human density data were obtained from an open-source database for spatial demography (www.worldpop.org) for 2011, 2014, 2016 at a resolution of 1 km. The densities of chicken farms and domestic duck farms of each study farm were extracted from a raster map constructed with the geographical coordinates of chicken and domestic duck farms provided by the Animal and Plant Quarantine Agency, Ministry of Agriculture, Food and Rural Affairs in South Korea in 2016 using the ArcGIS version 10.8.1 (ESRI Co., Redlands, CA, USA). An LBM is a place to sell live chickens or ducks and is known to play a major role in distributing viruses (31, 32). The minimum distance to the LBM from each farm was calculated using the geographical coordinates of 1,904 markets provided by the Ministry of Agriculture, Food and Rural Affairs in South Korea. In addition, geographical coordinates of the major wintering site for migratory wild bird habitats (195 sites, as of 2014) were obtained from the Ministry of Environment. Details on the geographic properties of the spatial variables used in this study can be found in Supplementary Figures S1–S12, the Supplementary Material.

### Investigation of Spatial Characteristics of HPAI-Infected Premises

Explanatory data analysis for 12 spatial variables was conducted on H5N1, H5N8, and H5N6 subtypes of HPAIv-infected chicken and duck holdings (88 IPs for H5N1, 339 IPs for H5N8, and 334 IPs for H5N6) to understand the spatial characteristics. An analysis of variance test or Kruskal–Wallis test, dependent on the normality of the variable to be tested, was performed to compare the mean of the spatial variables among the three subtypes of HPAIv-IPs (H5N1, H5N8, and H5N6), followed by a *post-hoc* analysis on each pair of IPs subtypes. The normality of spatial variables on a continuous scale was examined by the Shapiro-Wilk normality test and the Q-Q plot method. The significance level for the mean difference was set at *P* < 0.05. R software (version 4.1.2) was used for comparison tests.

### Construction of Prediction Model From a Case-Control Study on HPAI H5N8 Virus Epidemic

To identify spatial risk factors associated with HPAI incidence in poultry holdings, especially chicken and domestic duck farms, a case-control study was designed for the 2014–2016 HPAI H5N8 epidemic because it was the most prolonged nationwide outbreak (three waves with a total duration of 562 days) (28). A case farm was defined as the farms that raised either chicken or domestic duck and confirmed HPAI infection with a real-time reverse transcription-quantitative polymerase chain reaction test, according to the World Organization for Animal Health. The control group was selected from poultry farms that had (i) never experienced HPAI outbreak during the epidemic, (ii) a matching municipality with the case group, and (iii) species (i.e., chickens and domestic ducks) comparable to the case group, using a simple random sampling method with frequency matching at the same proportion for species and municipalities. Furthermore, farms for which location information was not available were excluded from the study. Finally, 339 HPAIv H5N8 positive poultry farms, consisting of 283 HPAI-positive duck farms and 56 chicken farms as the case group, and 603 negative poultry farms, which comprised 514 duck farms and 112 chicken farms, were selected as the control group.

Univariate analysis used a significance level of 0.05 to validate the mean difference of given variables between the case and control groups. Correlations among spatial risk variables were examined to identify interaction and confounding variables, and variance inflation factors (VIFs) were estimated to check the degree of multicollinearity. Consequently, the variables were excluded from the logistic analysis if the value of VIF value exceeded 3 (33). Subsequently, Bayesian multiple logistic regression analysis was applied to statistically significant variables with a *P* < 0.2 in univariate analysis (34). Furthermore, a multiple logistic regression model was built for either chicken or domestic holdings to identify spatial risk factors associated with HPAI occurrences at each species farm. The Gelman–Rubin convergence diagnostic was used to check the MCMC convergence of marginal posterior distribution of the regression coefficient estimates. The Morans‘I test on the residual from the Bayesian model was performed to check whether the spatial autocorrelation was presented in the data using 999 simulations. R2JAGS R package version 0.7.1 and ape R package version 5.6 were used for Bayesian regression analysis and Morans‘I test, respectively (35, 36).

Finally, the prediction model using the Bayesian multiple logistic regression analysis was built from the case-control study on the H5N8 epidemic with significant variables and non-significant associations because of the generalization of the model to predict other epidemics where other variables could be contributable to HPAI infection at poultry holdings. In addition to statistical approaches to predict other subtypes of HPAI outbreaks, the extreme gradient boosting (XGBoost) model was constructed using the same case and control farms of H5N8 epidemic used in the Bayesian multiple logistic regression analysis. Then, the XGBoost model predicted the probability of H5N1 and H5N6 subtypes of HPAI occurrence. XGBoost is a tree-based method used to model nonlinear relationships using the boosting technique to adaptively combine a large number of relatively simple tree models with improving predictive performance. Boosting refers to an ensemble method in which sets of models are trained sequentially, with each model learning from the errors of its predecessor, transforming a weak classifier into a strong classifier using weights. In addition, the XGBoost algorithm uses a regularized loss function to minimize overfitting. This diminishes the effect of a single tree on the final score by scaling down the weight of each new tree. Furthermore, it uses a variable selection approach to build a tree (37). In the XGBoost model, the data should include the presence and absence of HPAI in poultry farms for binary classification. We trained the model using hyperparameters under default settings. Bayesian logistic regression model fits were assessed using the Deviance information criterion (DIC) value using the stepwise method. Finally, the fit of the XGBoost model was assessed using 5-fold cross-validation. The R software version 1.5.0.2 xgboost package was used for XGBoost modeling (38).

### Prediction of the H5N8 and H5N6 Subtype Outbreak in Poultry Farms and Risk Mapping

We predicted the probability of incidence for other subtypes of HPAI in chicken and domestic duck holdings across the nation. We used 88 H5N1 IPs, 335 H5N6 IPs, 270 non-IPs, and 335 non-IPs randomly selected during each corresponding epidemic (see Supplementary Figure S13 in the Supplementary Materials) using two different methodological approaches (Bayesian logistic model and XGBoost model). The predictive performance of the Bayesian logistic model and XGBoost model on the three subtypes of HPAI virus infection was estimated using the area under the curve (AUC) of the receiver operating characteristic (ROC) curve. Moreover, the sensitivity and specificity of the model were calculated using the optimal cutoff that maximizes the distance to the diagonal line of ROC curve. Mapping and geographical visualization were performed using ArcGIS version 10.8.1. The HPAI risk map was constructed using the empirical Bayesian kriging interpolation method based on the probability of HPAIV incidence predicted by the Bayesian logistic regression model and XGBoost model using ArcGIS 10.8.1 software. The pROC R package version 1.18.0 was used to calculate the AUC, sensitivity, and specificity (39).

## Results

### Overview of Spatial Characteristics of the Three Subtypes Virus IPs

Table 3 provides a statistical summary of 12 spatial variables among the H5N1, H5N8, and H5N6 subtypes of HPAIv IPs. Overall, there were distinctive heterogeneities in the distribution of all spatial variables except for the proportion of wetland within a 3 km radius and the minimum distance to major wintering sites for wild birds. However, comparing the distributions of spatial variables between two pairs of subtypes of HPAIv IPs (H5N1-H5N8, H5N1-H5N6, and H5N8-H5N6) by a *post-hoc* analysis revealed similar characteristics in the combination of pairs (Supplementary Figure S14, Supplementary Materials). For example, the mean difference of the minimum distance to LBM between H5N1 and H5N8 IPs, H5N8, and H5N6, was not significantly different. Similarly, H5N1 and H5N6 IPs had comparable densities of chicken and domestic duck farms.

**Table 3:** Overview of spatial variables among three subtypes of highly pathogenic avian influenza virus infected premises (H5N1, H5N8, and H56) in South Korea.

	**Subtype**			
**Variables (unit)**	**H5N1 (*n* = 88)**	**H5N8** **(*n* = 339)**	**H5N6** **(*n* = 335)**	***p*-value**
Farm species				
Chicken (no. of farms)	34	56	197	-
Domestic duck (no. of farms)	54	283	138	-
Topology				
Elevation (m)	54.08 (42.03)	50.15 (41.68)	71.03 (49.14)	<0.01*^‡^
Topological wetness index^†^	10.45 (1.37)	11.07 (1.83)	10.63 (1.59)	<0.01*^‡^
Land-use/cover				
Proportion of forest within a 3 km radius (%)	25.67 (20.29)	21.08 (17.21)	29.45 (19.47)	<0.01*^‡^
Proportion of rice field within a 3 km radius (%)	33.58 (14.81)	37.33 (11.29)	32.09 (13.15)	<0.01*^‡^
Proportion of waterbody within a 3 km radius (%)	2.98 (2.73)	2.44 (1.63)	2.77 (1.80)	<0.01*^‡^
Proportion of wetland within a 3 km radius (%)	1.13 (1.09)	0.97 (0.89)	1.22 (1.89)	0.07^‡^
Minimum distance to driveway (km)	0.53 (0.47)	0.59 (0.51)	0.43 (0.41)	<0.01*^‡^
Poultry and human				
Human (no. of inhabitant/10 km^2^)	30.40 (42.02)	17.69 (19.61)	31.768 (39.74)	<0.01*^§^
Chicken farm (no. of farms/km^2^)	0.34 (0.27)	0.60 (1.20)	0.62 (0.76)	<0.01*^§^
Domestic duck farm (no. of farms/km^2^)	0.32 (0.37)	0.89 (1.13)	0.51 (0.97)	<0.01*^§^
Wildlife and live bird market				
Minimum distance to major wintering site for wild bird (km)	5.55 (5.77)	6.32 (5.56)	6.59 (4.45)	0.23^‡^
Minimum distance to live bird market (100 m)	1.04 (0.10)	3.10 (12.8)	2.41 (9.24)	<0.04*^§^

*Values are expressed as mean (standard deviation)*.

† *Topological wetness index indicates the level of water accumulation and local drainage*.

‡ *Using ANOVA test*.

§ *Using Kruskal–Wallis test*.

More specifically, H5N8 IPs were mainly composed of domestic duck farms (83.5% of total study farms), at lower elevations with a spacious rice field, very low human density, and higher domestic duck farm density than other subtypes of HPAI virus IPs. In contrast, H5N6 IPs with a higher proportion of chicken farms (58.8% of the total study farms) were placed at higher elevations with higher forest area, higher human, and chicken farm density, and closer to driveways. Additionally, H5N1 IPs, similar proportion in farm species, located in the medium range of elevations between H5N8 and H5N6 IPs, had the lowest chicken and duck farm density with the lowest minimum distance to the wintering site for wild birds and the LBM.

### Identification of Spatial Risk Factor in the H5N8 Subtype Outbreak

Table 4 summarizes the adjusted odds ratio of spatial variables in the Bayesian multivariate logistic regression analysis on both chicken and duck farms, following univariate logistic regression analysis for screening possible associated variables (see Supplementary Tables S1–S3). The proximity to LBM was identified as a spatial risk factor of H5N8 occurrence in poultry farms. Indeed, chicken and duck farms closer to LBM had a higher risk of HPAI during the H5N8 epidemic.

**Table 4:** Summary of marginal posterior distribution of adjusted odds ratio of spatial variables in the Bayesian multivariate logistic regression for H5N8 outbreaks.

**Variable**	**Mean (95% Crls)**
Topological wetness index	1.058 (0.952, 1.182)
Proportion of forest	0.993 (0.972, 1.014)
Proportion of rice field	0.988 (0.960, 1.016)
Minimum distance to driveway	1.163 (0.764, 1.769)
Human density	0.963 (0.782, 1.144)
Domestic duck farm density	1.940 (1.488, 2.624)
Minimum distance to major wintering site for wild bird	1.025 (0.973, 1.079)
Minimum distance to LBM	0.469 (0.457, 0.500)
DIC	682.645
AUC	0.929
Morans‘I (*p*-value)	0.124 (0.281)

*LBM, live bird market; DIC, deviance information criterion; AUC, area under the curve*.

**Figure 3:**
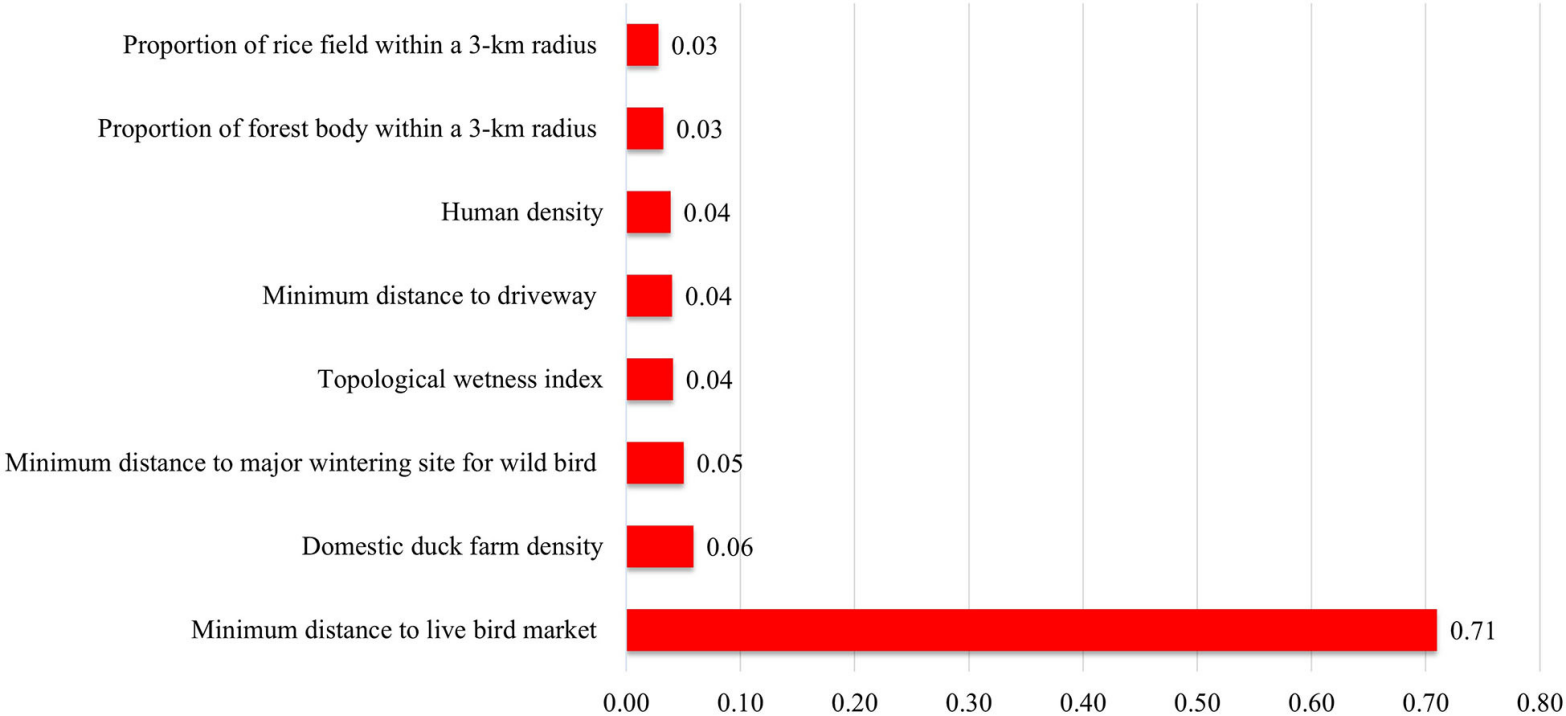
Importance of spatial variables used in the XGBoost model. The value of importance represents the relative contribution of the corresponding variable to the prediction of the observed results in the model ranging from zero to one.

More specifically, for the adjusted odds ratio of spatial variables in both chicken and duck farms, a higher domestic duck farm density and lower minimum distance to the LBM contributed to H5N8 occurrence in farms of these species. When comparing case and control farms producing only chicken, a higher chicken farm density and lower minimum distance to LBM significantly increased the risk for HPAI occurrence (see Supplementary Table S4). The marginal posterior distribution of regression coefficients was converged with the Gelman-Rubin index of < 1.01 (see Supplementary Figure S15). Additionally, in training the data consisting of eight spatial variables and a response binary variable for HPAI infection using XGBoost, the minimum distance to the LBM was the most important factor, accounting for approximately 71% of the variation in the model, followed by domestic duck farm density as shown in Figure 3. Duck farm density showed an increasingly positive correlation with HPAI occurrence of 1.5 farms /km^2^, whereas the minimum distance to LBM had a negative correlation, which decreased to zero at a point of 1.3 km. For more details on the dependence between a set of spatial variables and the HPAI outbreak, refer to Supplementary Figure S16.

### H5N8 and H5N6 Subtype Outbreak Prediction and Risk Mapping

Table 5 presents the predictive performance of the Bayesian logistic regression and XGBoost models for H5N1, H5N8, and H5N6 in chicken and domestic duck holdings. With the model built based on a case-control study of the H5N8 epidemic, two models generally showed high predictive performance for different subtypes of HPAI occurrence in poultry farms. However, the machine learning approach (XGBoost) outperformed the statistical approach (Bayesian logistic regression) in predicting all three subtypes of HPAI occurrences. For example, the XGBoost model reported an AUC of > 0.968 for all types of prediction, whereas the Bayesian regression model exhibited an AUC of > 0.818. Moreover, there was a pronounced difference in prediction sensitivity between the two models. For predicting the H5N1 outbreaks, the XGBoost model reported a sensitivity of 1.00, whereas the Bayesian regression model showed a sensitivity of 0.615.

**Table 5:** Predictive performance of two different methodological approaches on three subtypes of highly pathogenic avian influenza outbreaks in poultry farms.

**Model**	**Bayesian logistic regression**				**Machine learning algorithm (XGBoost)**			
**Subtype**	**AUC**	**Sensitivity**	**Specificity**	**Cutoff**	**AUC**	**Sensitivity**	**Specificity**	**Cutoff**
H5N1	0.818	0.615	0.930	0.285	0.995	1.000	0.980	0.884
H5N8	0.929	0.912	0.851	0.502	0.996	0.973	0.961	0.445
H5N6	0.887	0.803	0.815	0.005	0.968	0.928	0.943	0.239

*Sensitivity and specificity are calculated based on the best cut-off*.

*AUC, area under curve*.

**Figure 4:**
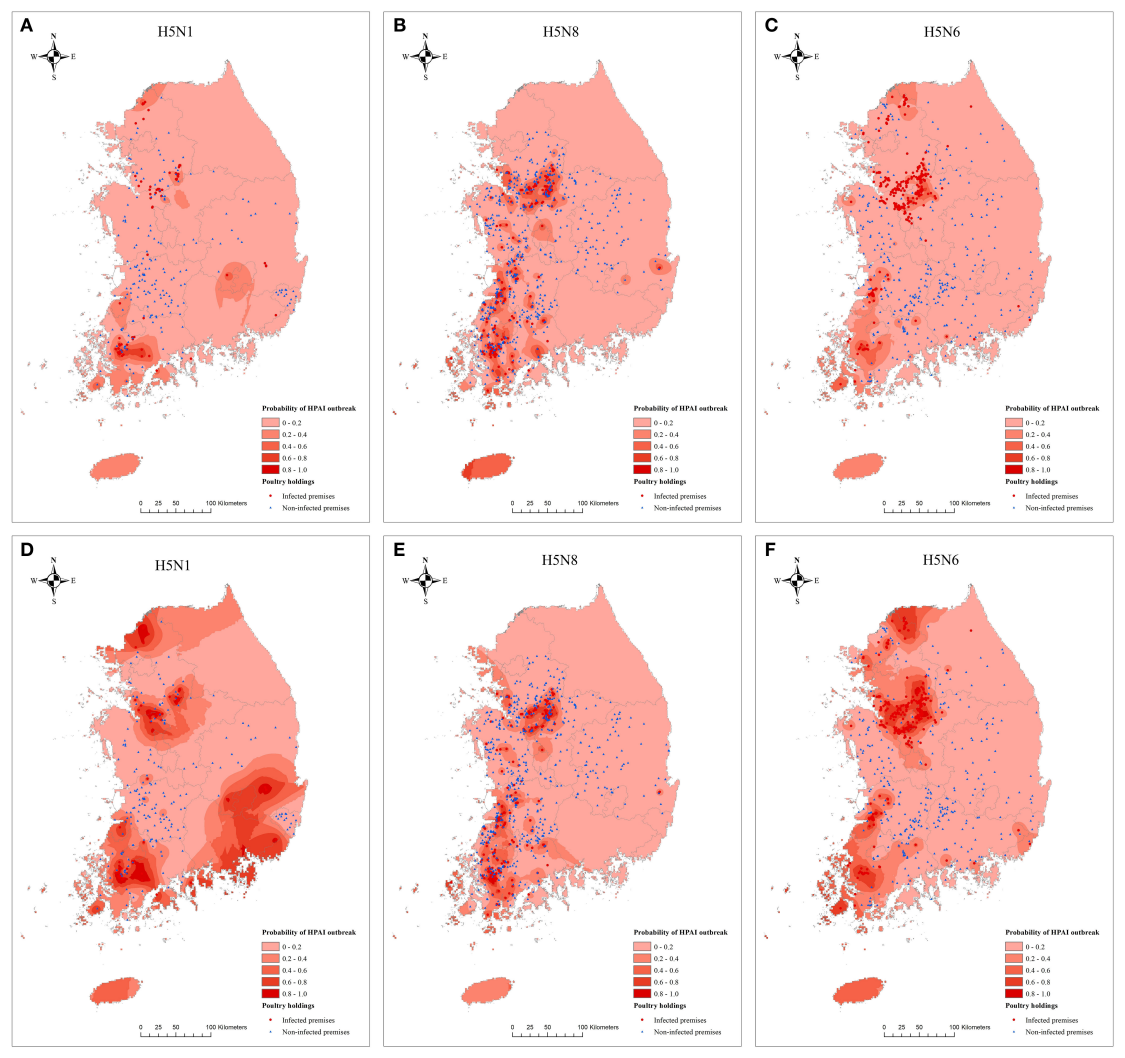
Risk map predicted by Bayesian logistic regression model for H5N1 **(A)**, H5N8 **(B)**, and H5N6 **(C)** and XGBoost model for H5N1 **(D)**, H5N8 **(E)**, and H5N6 **(F)** of highly pathogenic avian influenza outbreaks in poultry holdings.

Geographically, both models consistently predicted a higher probability of HPAI occurrence in Naju, Yeongam, and Suncheon, which are the southwestern parts, and Anseong, Cheonan, and Eumseong, which are the central parts of the nation, irrespective of the subtype (see Figure 4). However, the risk map generated using the XGBoost showed higher risk in the southeastern area during the H5N1 epidemic and in the northern area during the H5N6 epidemic than those of the Bayesian regression model.

## Discussion

With the expectance of the emergence of novel HPAIv, it is imperative to precisely predict the risk of HPAI occurrence among poultry farms to implement proper biosecurity measures. South Korea is one of the countries under the area at high risk of HPAI occurrence, in the particular winter season, with massive damages recently even worsening due to the geographical location adjacent to endemic countries, part of East Asian–Australasian Flyway, the intensification of poultry production. Unfortunately, animal health and production authorities in South Korea have limited knowledge about the common risk factors related to various subtypes of HPAI epidemics in poultry farms from a spatial perspective. Therefore, this study aimed to identify spatial risk factors associated with HPAI infection in poultry farms, as discussed in previous studies in many other countries, and subsequently predict the probability of infection in poultry farms at high risk in order to prevent HPAIV occurrence in the near future.

In this study, the three subtypes of HPAIv-IPs exhibited geographically heterogeneous characteristics, although similarities between the pairs of different subtypes of the outbreaks were often observed. In particular, land use surrounding IPs varied across the subtypes of the HPAI epidemic. This can be partly explained by the difference in composition of species in infected farms, which is closely related to the demographic and economic conditions in South Korea. The domestic duck farms in South Korea are concentrated in relatively small areas, especially in the southwestern region, which produced 48% of total ducks in 12,247 square kilometers according to the Korean National Agriculture Statistics, but chicken farms are evenly distributed throughout the country.

Unlike domestic duck farms, most HPAI cases in chicken farms are reported in layer farms, which appear to require far more labor force than domestic duck farms because of a variety of specialized work, including cage cleaning, egg collection, and delivery, which is attributed to its location close to human populated areas where people are easily recruited (30). Also, the supply chain facilitates the location of layer farms that are close to the human population for egg sales (24). Therefore, domestic duck farms, which were the predominant type of infected farm species during the H5N8 epidemic, are more likely to be surrounded by a high proportion of rice fields within a 3-km radius of a poultry farm. In contrast, H5N1 and H5N6 IPs, which were comparably composed of duck and chicken farms, were located within a relatively higher human density along with a lower proportion of rice fields.

Additionally, governmental intervention strategies driven by the transmision pathway during an epidemic could affect the disparity in spatial characheristics. For example, during the 2010–2011 H5N1 epidemic, the trade through the LBM, was not prohibited and became the major source of infection with pathogens. After numerous HPAI infection cases were found to be epidemiologically linked to the LBM, the government rapidly imposed heavy restriction on live bird sales, such as the temporary closure of LBM during the H5N8 and H5N6 epidemic. Consequently, at higher value for the minimum distance to the LBM from IPs during theses epidemics was observed compared with H5N1 IPs.

In a case-control study of the HPAI H5N8 epidemics, duck farm density and minimum distance to the LBM were found to be major predisposing factors for HPAI infection in poultry farms. The density of domestic duck, as the previous study reported that there was a strong association between the density of ducks and HPAI outbreak farms, was also identified as an important risk factor of HPAI in Korea in this study (40, 41). Generally, domestic ducks in South Korea are kept indoors in a shed made with vinyl and temporary plastic panels, comparted with chicken, resulting in very low biosecurity systems and hygiene levels, even in places where there is no special facility installed for cleaning and disinfection because of economic disadvantage (42).

Moreover, all-in-all-out production practices are not usually adapted to domestic duck farms, where day-old ducklings were introduced into the farms continuously without the least downtime (28) because 95% of farms contracted poultry integrators which are likely to assure income security by producing certain number of ducks into the market (43).

Additionally, meat ducks that account for more than 90% of ducks in South Korea have a rearing period of 45 to 48 days, longer than the breeding period of meat chickens (21 to 27 days), which led to longer HPAIv periods (6). This longer rearing period could make domestic duck farms vulnerable to exposure to vehicles and medium contaminated with viruses shed from infected birds and poultry. In addition, domestic duck shows less severe clinical signs and sheds HPAIv longer than chickens (44). Therefore, the risk of HPAIv transmission between domestic duck farms in areas with a high density of duck farms escalates after virus incursion in the area without being timely detected in domestic ducks, which increases the risk of HPAI infection in other farms through local spread (43). Historically, the southwestern areas in South Korea, where a high proportion of IPs were domestic duck farms, are agricultural regions, producing a diverse range of ingredients for food, becoming a popular place for food (45), where abundant LBMs and numerous restaurants specialized in duck meat cuisine are up and running (46). In addition, due to tourists' and locals' preference for fresh meat duck dishes, small-scale duck sale and trade was actively conducted through LBM, where no strict biosecurity system and surveillance program were generally administered (31). Consequently, it is plausible that the infected duck farms in these areas are geographically close to the LBM.

The Bayesian logistic regression and XGBoost model built based on the H5N8 case-control study demonstrated a relatively high predictive performance (AUC value > 0.8) for retrospective (H5N1) and prospective epidemics (H5N6), although they outperformed the risk prediction for H5N8 outbreak. This suggests that although the overall spatial characteristics of IPs among the three subtypes of epidemics showed disparity to some extent, the distributions of spatial variables of IPs and non-IPs during the H5N1 and H5N6 epidemics were distinguishable to clarify either HPAI infection or non-infection. Moreover, significant key variables of those IPs, such as the minimum distance to the LBM, had enough values to give weights to the model for a higher probability of HPAI infection. For instance, the mean of the minimum distance to the LBM for H5N1 IPs and H5N6 IPs was 1.04 and 2.41, respectively. This value is lower than H5N8 IPs, leading to higher odds of HPAI infection in the logistic model and probability in the XGBoost model. Additionally, the machine learning algorithm model demonstrated higher predictive performance than the statistical model, especially for H5N1 and H5N6. The XGBoost is a tree-based approach that uses the regularized method, where the number of splits and depth in the decision tree is restricted by defining the minimum entropy gain necessary to create further splits (37). Indeed, the more splits or partitions for the classification rule for predictors, the more likely the model is to overfit the training data (47). Thus, this regularized method functions to prevent the model from overfitting to the training dataset; in this case, H5N8. Therefore, it produced comparable predictive performance in the test dataset, including H5N1 and H5N6.

In addition, from a geographical perspective on the distribution of poultry holdings at high risk, two regions, the central and southwest part of the country, were constantly predicted by the two models to have a higher probability of infection, which was consistent with the observed data. South Korea geographically has spacious land with plenty number of lakes, streams, and rivers in the western part and mountainous areas in the eastern part. The HPAI H5N8 outbreak in 2014 occurred in the southwestern part of South Korea in the early stages of the epidemic, with a large number of infections and continued in the eastern and central parts of the country. Similarly, the HPAI H5N1 and H5N6 outbreaks began in the central part of the country, spread to the eastern part of South Korea, but mostly occurred in the southwestern part, especially Yeongam, and Naju. This suggests that the occurrence of HPAI is largely affected by the biological characteristic of HPAIv subtype and the spatial characteristics associated with the production system and market chain structure. This is also supported because H5N1 and H5N6 occur in similar areas and farms where HPAI recurrences appear even though ownership and trading affiliates change.

It is important to consider addressing this issue in future work. First, we used poultry density data from 2016; however, according to the national livestock statistics, from 2011 to 2017, the number of duck and chicken farms slightly decreased, and new poultry farms rarely opened. However, depopulated farms started their production 6 months later, and density was presumed to remain similar across the study periods. Additionally, we chose the spatial variables based on the previous studies with a lack of elucidation of complex interactions among those variables. Thus, it is needed to build the prediction model based on the selection of variables from an understanding of the causal relationship of the variables with HPAI infection, such as a directed acyclic graph. Moreover, we used spatial data from diverse sources inherently with different spatial resolutions due to a diversity of spatial variables. Basically, the higher the spatial resolution of data for the corresponding spatial variables is, the closer the extracted value of the observation is to the true one. Therefore, we derived the value of corresponding spatial variables from the local data source, which provided a higher spatial resolution to minimize the deviation between the extracted and true value of spatial variables for study farms. Some variables extracted from a lower resolution, such as human density, where local data sources were not available, are likely to deviate from the true value. And the study farm within the cell of the spatial resolution had an identical value to the given variables. However, the 1 km spatial resolution (30 arc-second) was still considered to be high spatial resolution (46). Thus, given the characteristics of variables at 1 km spatial resolution, the estimation of the variable from the data source is not significantly biased. Secondly, although this study placed more weight on spatial variables to identify the association with HPAI and predict its risk in other epidemics, the on-farm biosecurity level at poultry holdings, such as adequate restriction to farm visibility, is a critical adjustment factor for estimating the direct influence of spatial variables on the risk of the HPAI outbreak. Therefore, future studies will also need to investigate biosecurity protocol compliance at the farm level to identify whether the association of spatial variables with HPAI infection in this study remains significant. Lastly, 48 farms out of H5N8 case farms were excluded from the case control study due to the missing information on their geographical location. All were small-scale farms (<50) or backyard farms that might hinder acquiring the exact information. For this reason, the model built from the H5N8 case and control farms is likely to underestimate the risk factors associated with HPAI infection in small-scale farms and or backyard farm.

Nonetheless, this study was the first to investigate the spatial risk factors of all subtypes of the HPAIv in South Korea from 2010 to 2017 and predict the risk of occurrence at the farm level. The results of this study emphasize that the viral and spatial characteristics of the poultry farm play an essential role in the development of HPAI. Therefore, to prevent and prepare for future HPAI occurrence, it is important to study the spatial characteristics of existing virus-type farms to prepare for the introduction of new viruses and to establish livestock prevention and prevention strategies based on this. Therefore, it is necessary to formulate HPAI prevention and response strategies through risk-based surveillance.

## Data Availability Statement

The original contributions presented in the study are included in the article/Supplementary Material, further inquiries can be directed to the corresponding author.

## Author Contributions

D-sY and BC conceived and designed the study. D-sY acquired the data, performed data analyses, and wrote the original draft. KH and JK supported data collection. BC, KH, and JK edited the subsequent drafts. All authors read and approved the final manuscript.

## Conflict of Interest

The authors declare that the research was conducted in the absence of any commercial or financial relationships that could be construed as a potential conflict of interest.

## Publisher's Note

All claims expressed in this article are solely those of the authors and do not necessarily represent those of their affiliated organizations, or those of the publisher, the editors and the reviewers. Any product that may be evaluated in this article, or claim that may be made by its manufacturer, is not guaranteed or endorsed by the publisher.
