# Supplementary material for: Risk Prediction of Three Different Subtypes of Highly Pathogenic Avian Influenza Outbreaks in Poultry Farms: Based on Spatial Characteristics of Infected Premises in South Korea
Source: Front Vet Sci. 2022 May 31;9:897763. doi: 10.3389/fvets.2022.897763 (PMC9194674; doi:10.3389/fvets.2022.897763)
Supplement: Supplementary file 1 [file Data_Sheet_1.docx]

Supplementary Material

# Materials and methods

*Measurement of fit of the machine learning model to HPAI H5N8 data*

We used cross-validation to assess the fit of the XGBoost model to the observed H5N8 data. Cross-validation is a resampling method that partitions total data into train set and validation set on different iterations. First, a total data set of H5N8 case and control farms were randomly split into five separate data sets where four data sets were used to train the model, and one data set was used for testing. Then, this process was repeated 500 times to calculate misclassification error rate ((false positive+false negative)/total observations) as measures of fit of the model based on the results across the iterations. Misscalssification error rate was calculated as follows

| **Table S1** Data source for spatial variables used for risk assessment of highly pathogenic avian influenza | | | | | |
| --- | --- | --- | --- | --- | --- |
| Data | Source | Periods | Type | Variables | Note |
| Digital elevation model | USGS SRTM* | 2014 | Raster | Elevation  Heat load  Topological wetness index | 30-m resolution |
| Human | Worldpop | 2011, 2014, 2017 | Raster | Human density | 1-km resolution |
| Land use/cover | Ministry of environment, Republic of Korea | 2009, 2017 | Vector (polygon) | The size of waterbodies, rice field, wetland, and forest | 5-m resolution |
| Live bird market | Ministry agriculture, livestock, food and rural affairs, Republic of Korea | 2014 | Vector (point) | Distance to live bird market |  |
| National roads | National geographic information institute, Korea | 2011, 2014,2 017 | Vector (line) | Distance to driveway | 5-m resolution |
| Poultry farms | Korea animal health integration system | 2008, 2014, 2016 | Raster | Density of chicken farms, domestic duck farms | 100-m resolution |
| Wild bird habitats for wintering | Ministry of environment, Republic of Korea | 2014 | Vector (polygon) | Distance to major wintering sites |  |

USGS, United States Geographical Survey; SRTM, Shuttle Radar Topography Mission


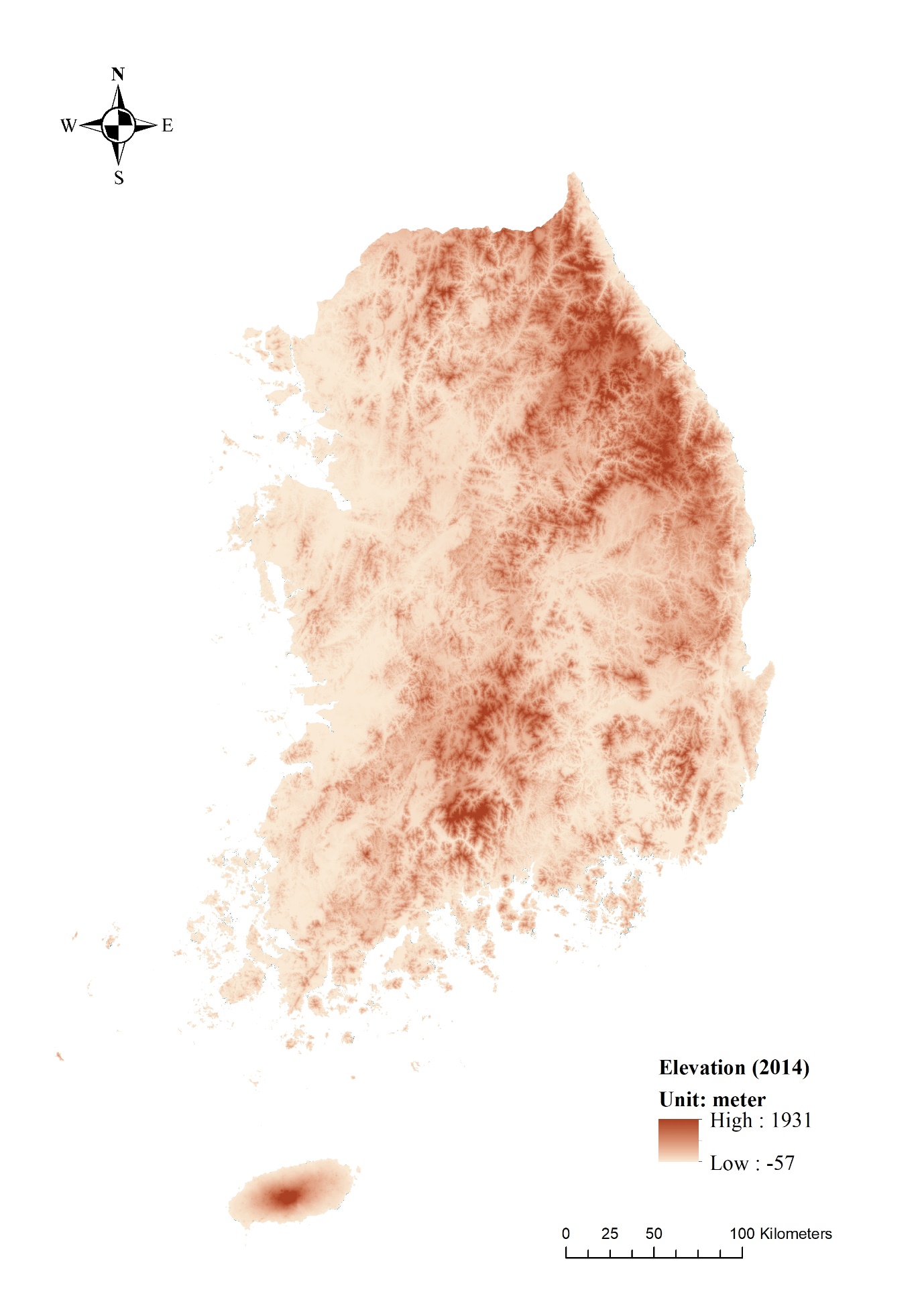


**Figure S1** Elevation of South Korea in 2014


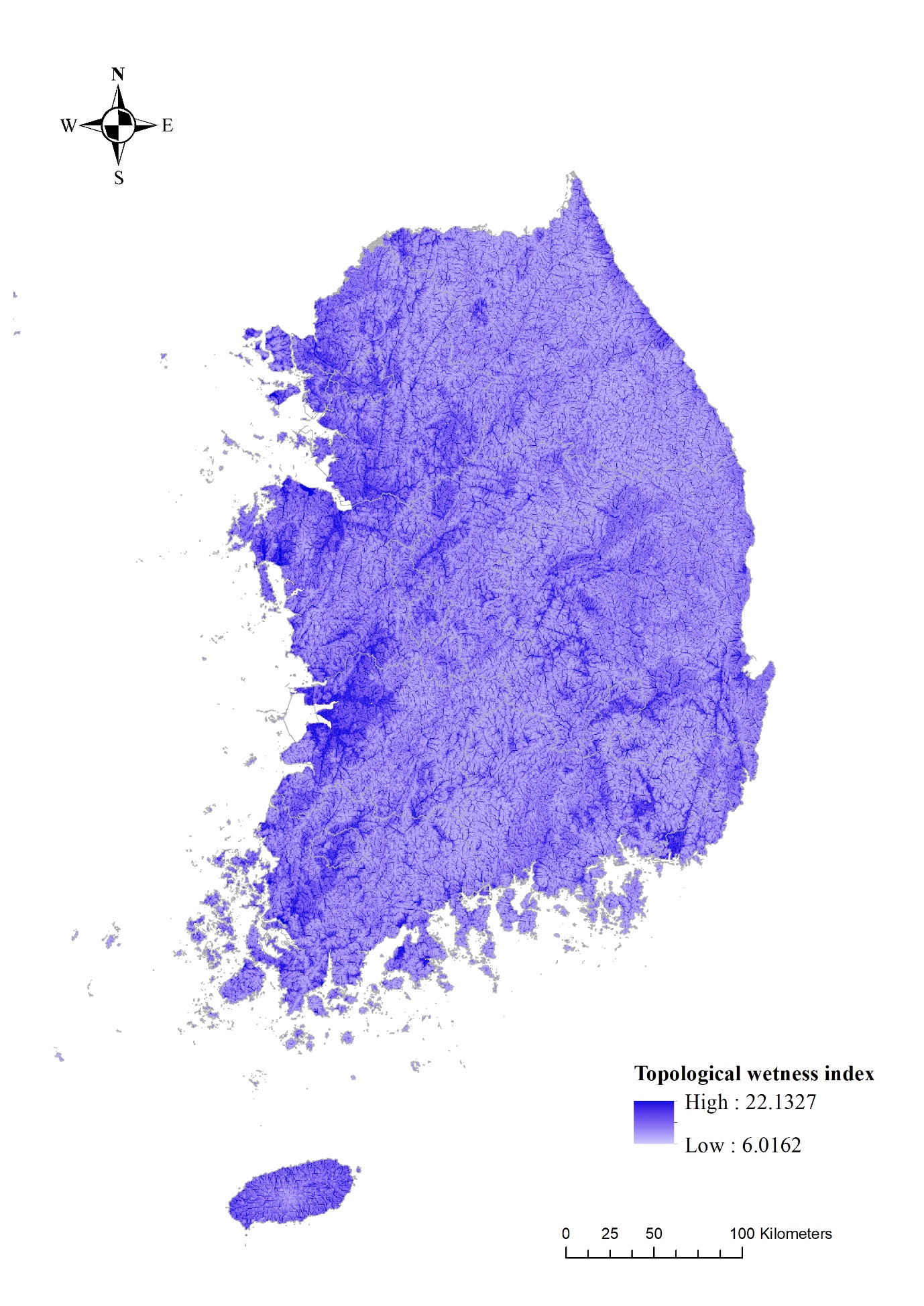


**Figure S2** Topological wetness index of South Korea in 2014


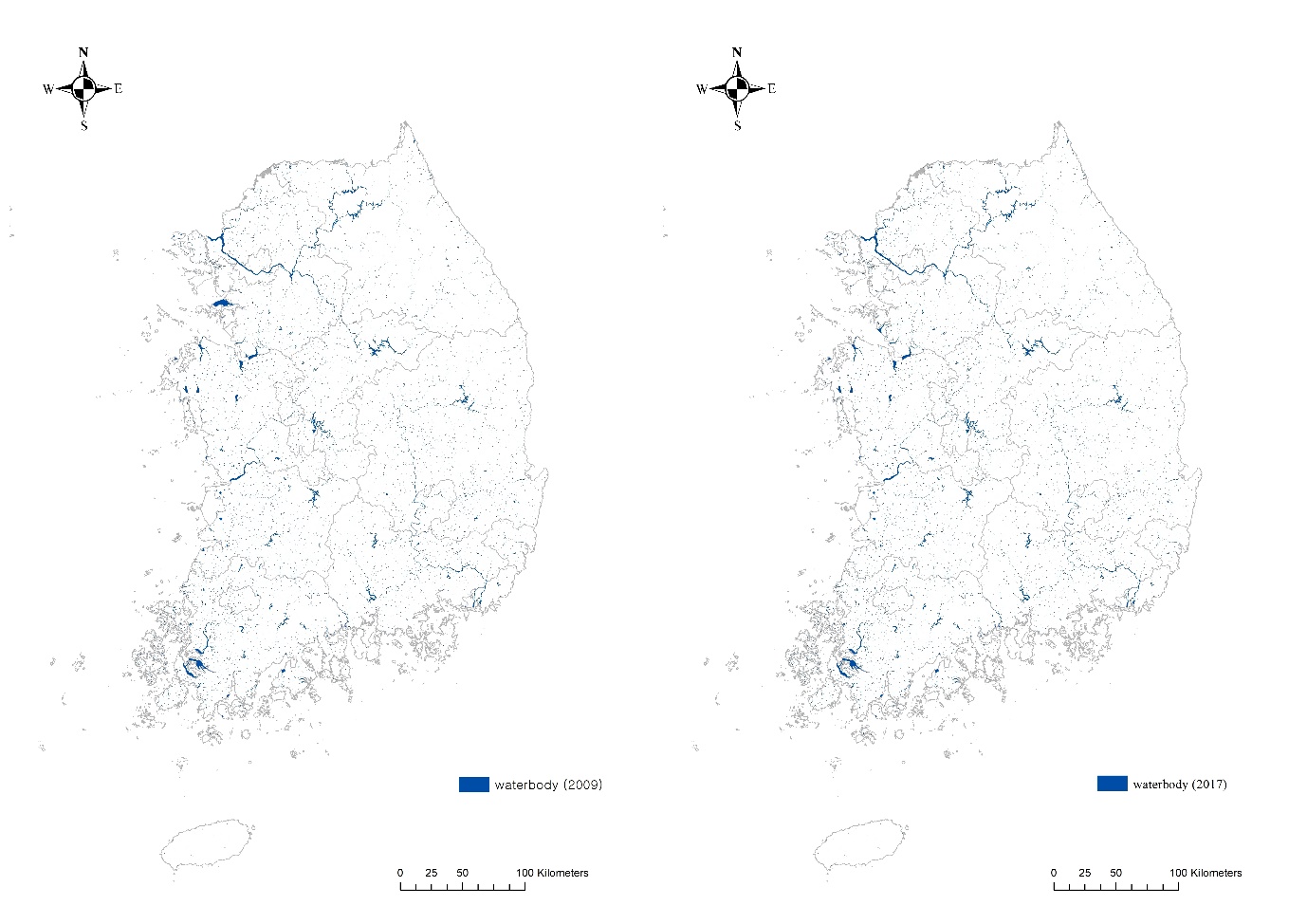


**Figure S3** Waterbodies in South Korea **(left in 2009 and right in 2017)**


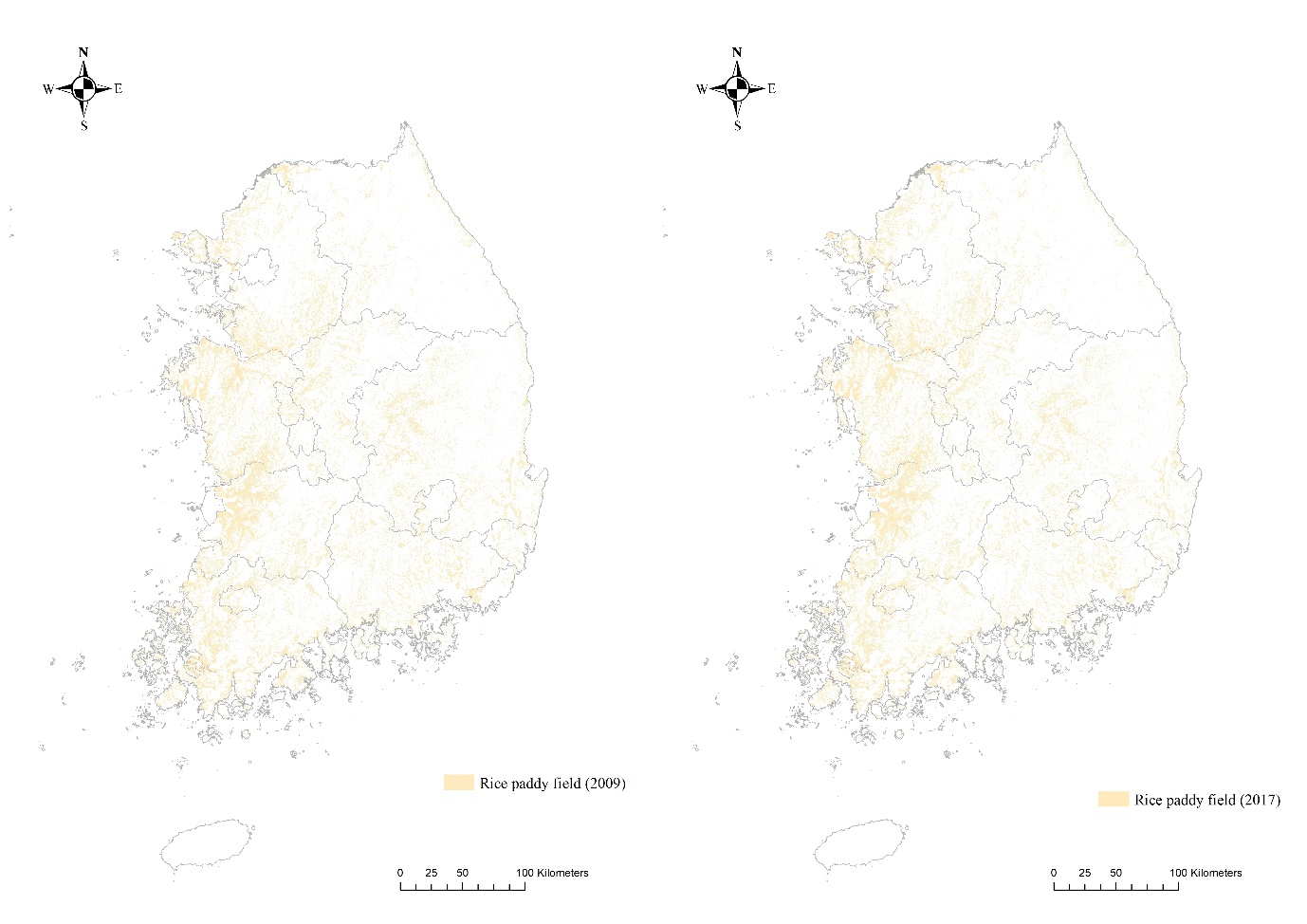


**Figure S4** Rice paddy fields in South Korea **(left in 2009 and right in 2017)**


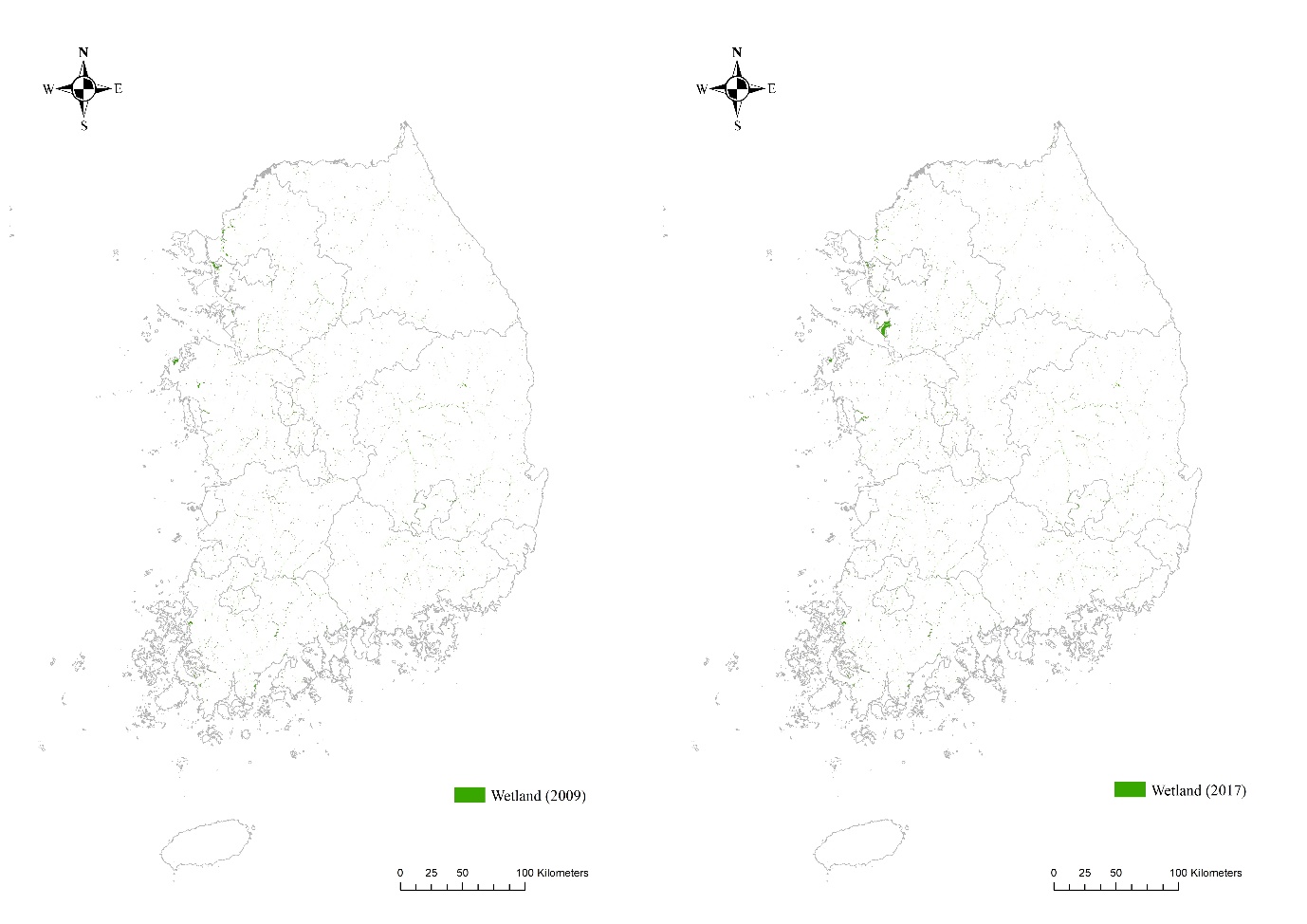


**Figure S5** Wetland in South Korea **(left in 2009 and right in 2017)**


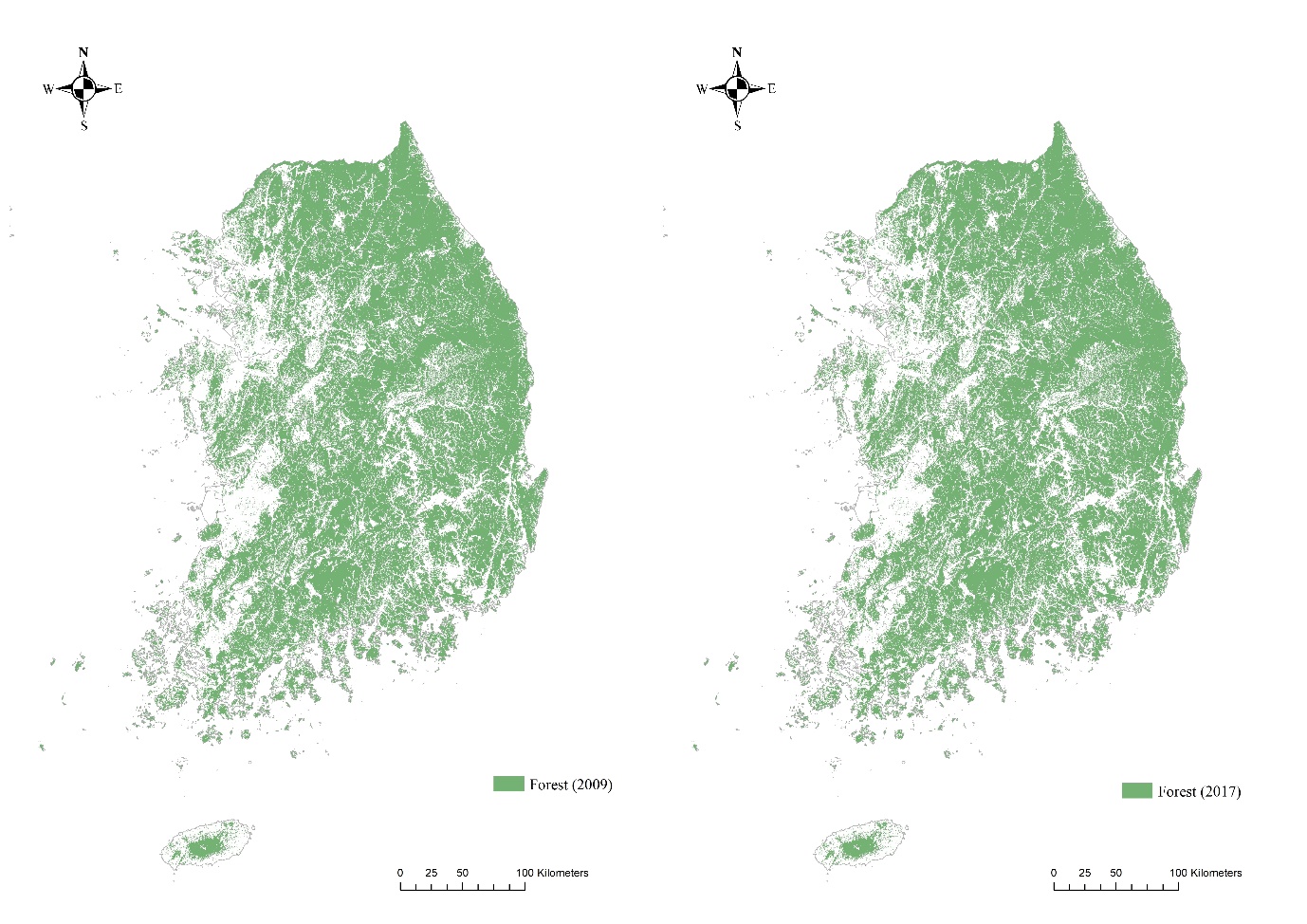


**Figure S6** Forest in South Korea **(left in 2009 and right in 2017)**


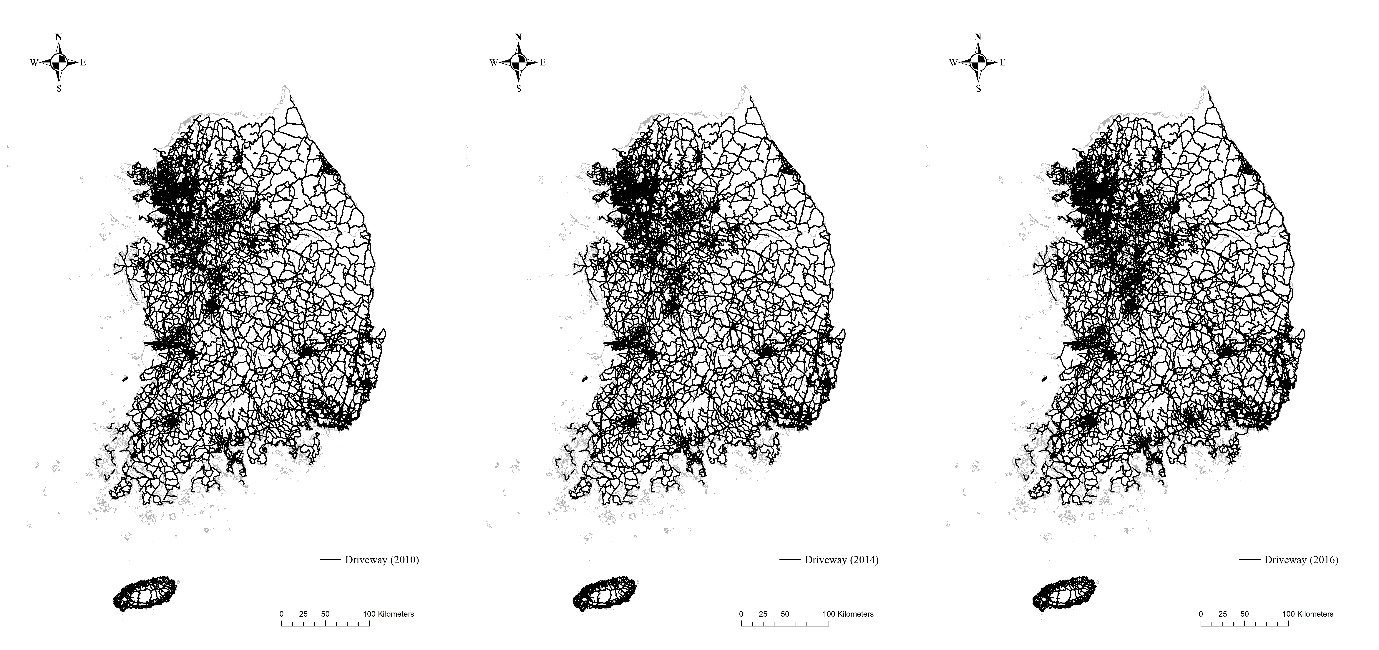


**Figure S7** Driveway in South Korea (**left in 2010, middle in 2014, and right in 2017)**


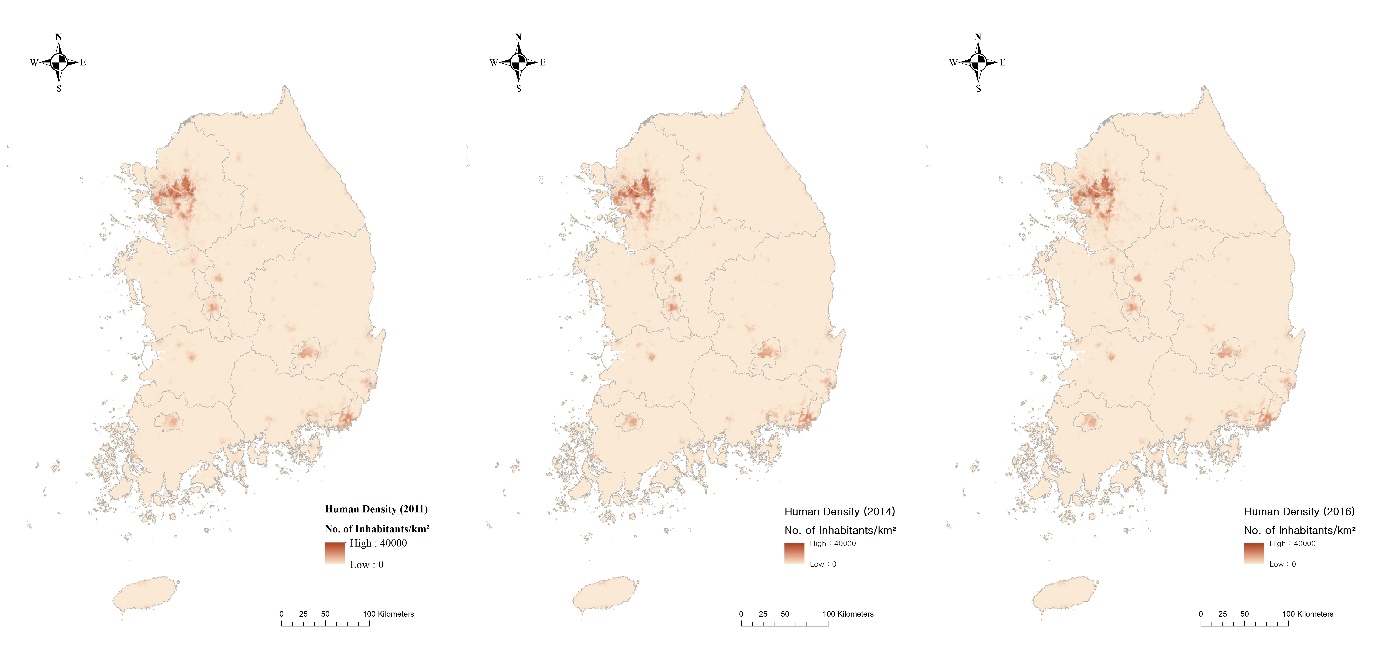


**Figure S8** Human density in South Korea **(left in 2010, middle in 2014, and right in 2017)**


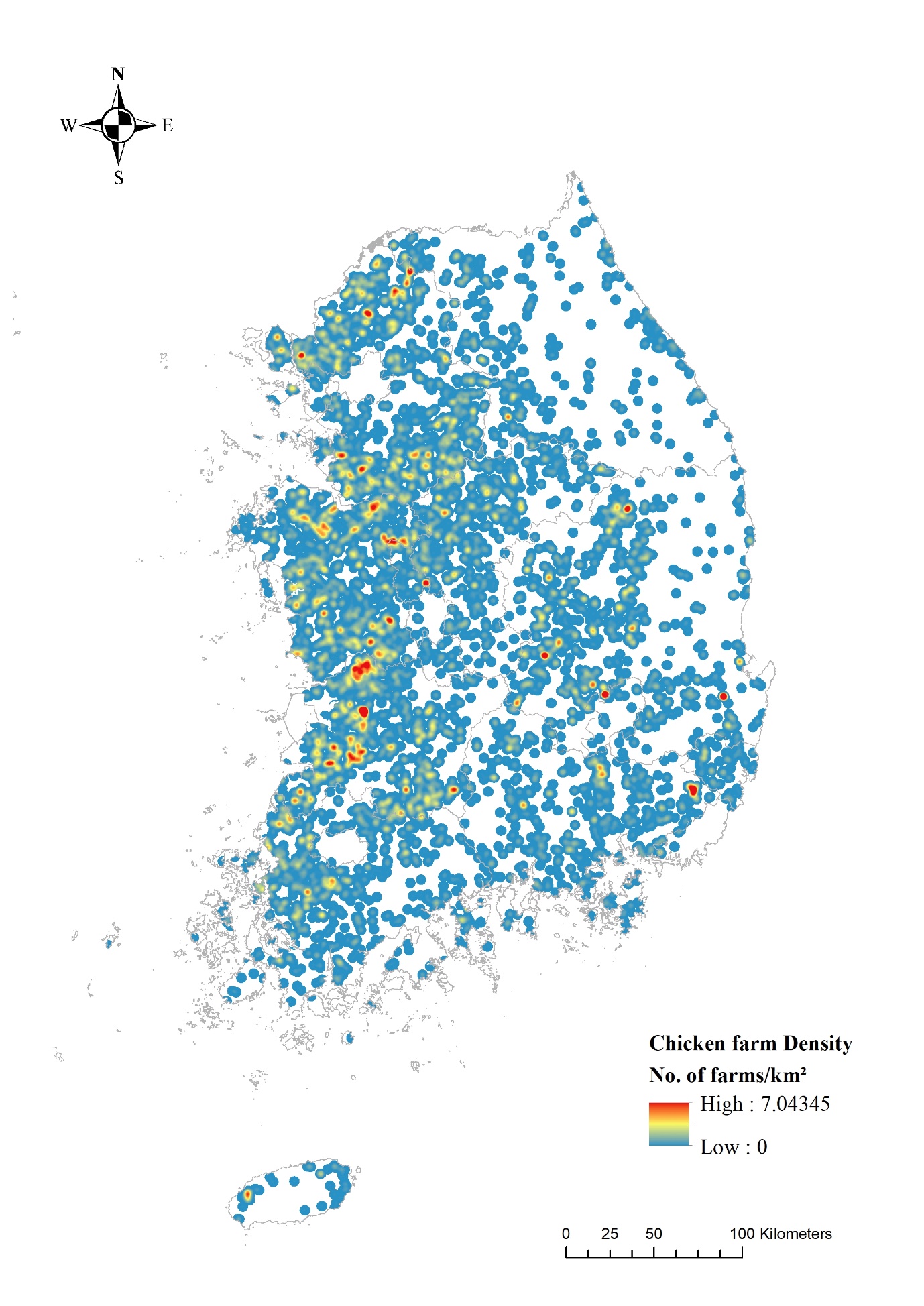


**Figure S9** Chicken farm density in South Korea in 2016


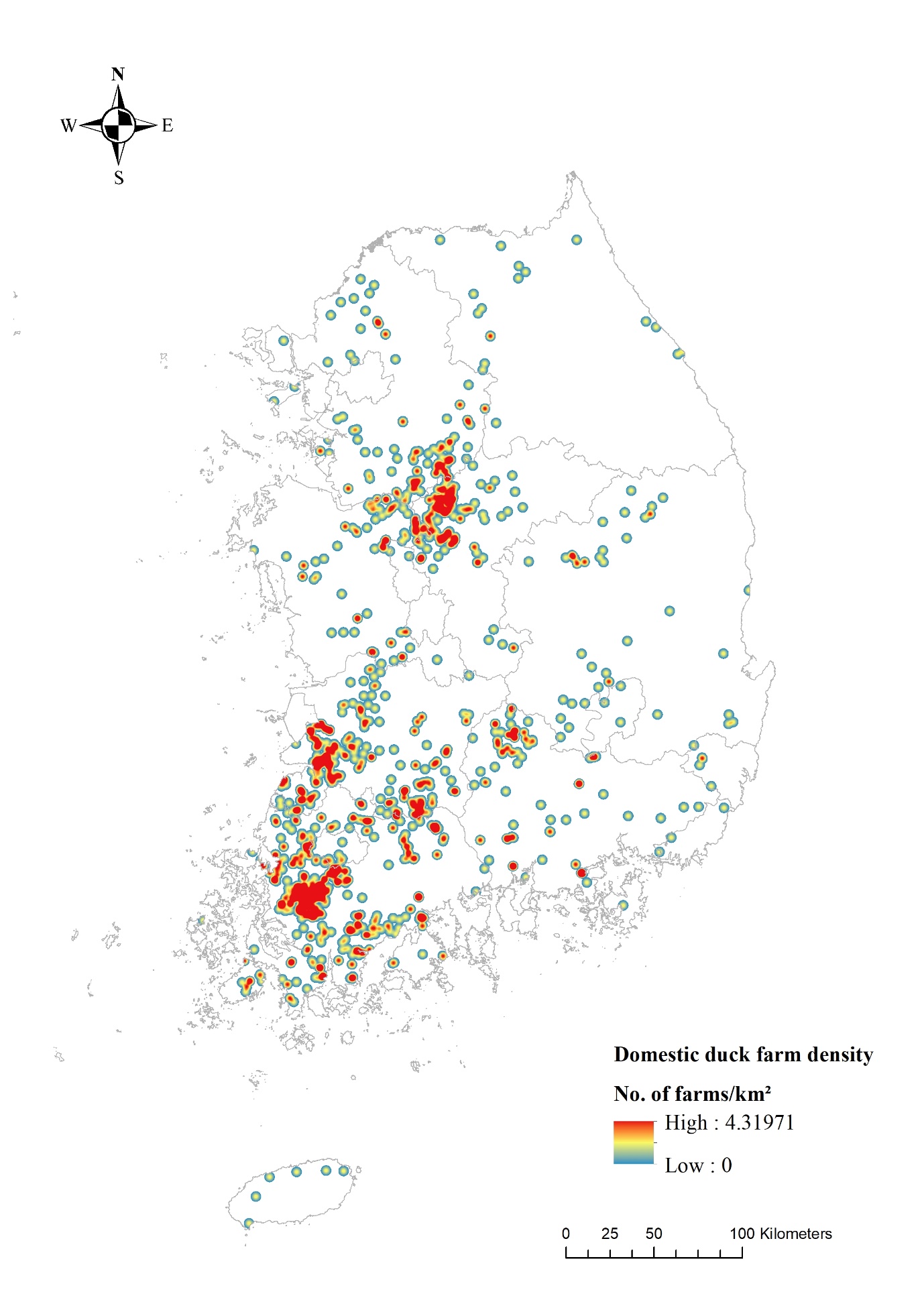


**Figure S10** Domestic duck farm density in South Korea in 2016


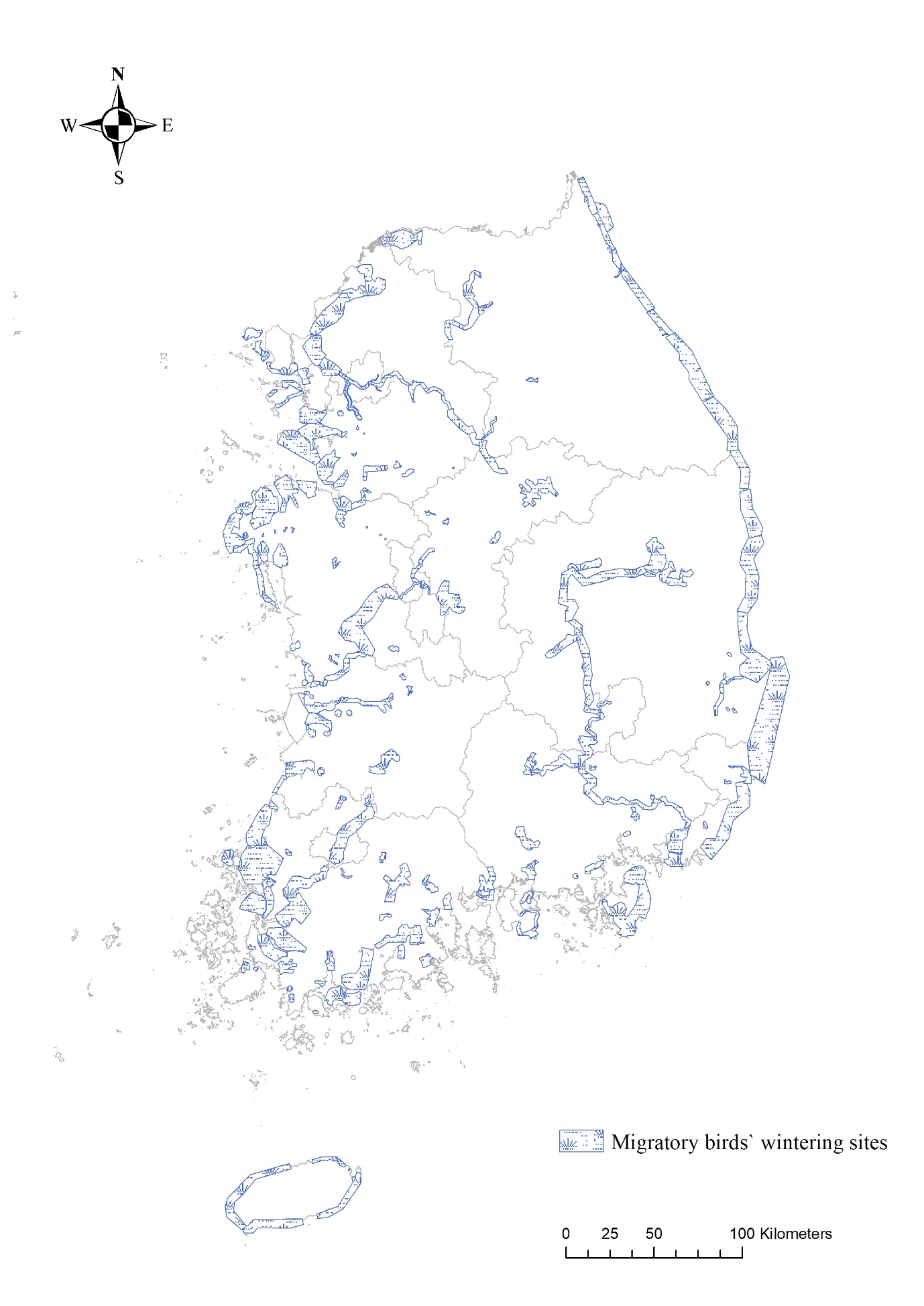


**Figure S11** Major migratory bird wintering sites in South Korea in 2016


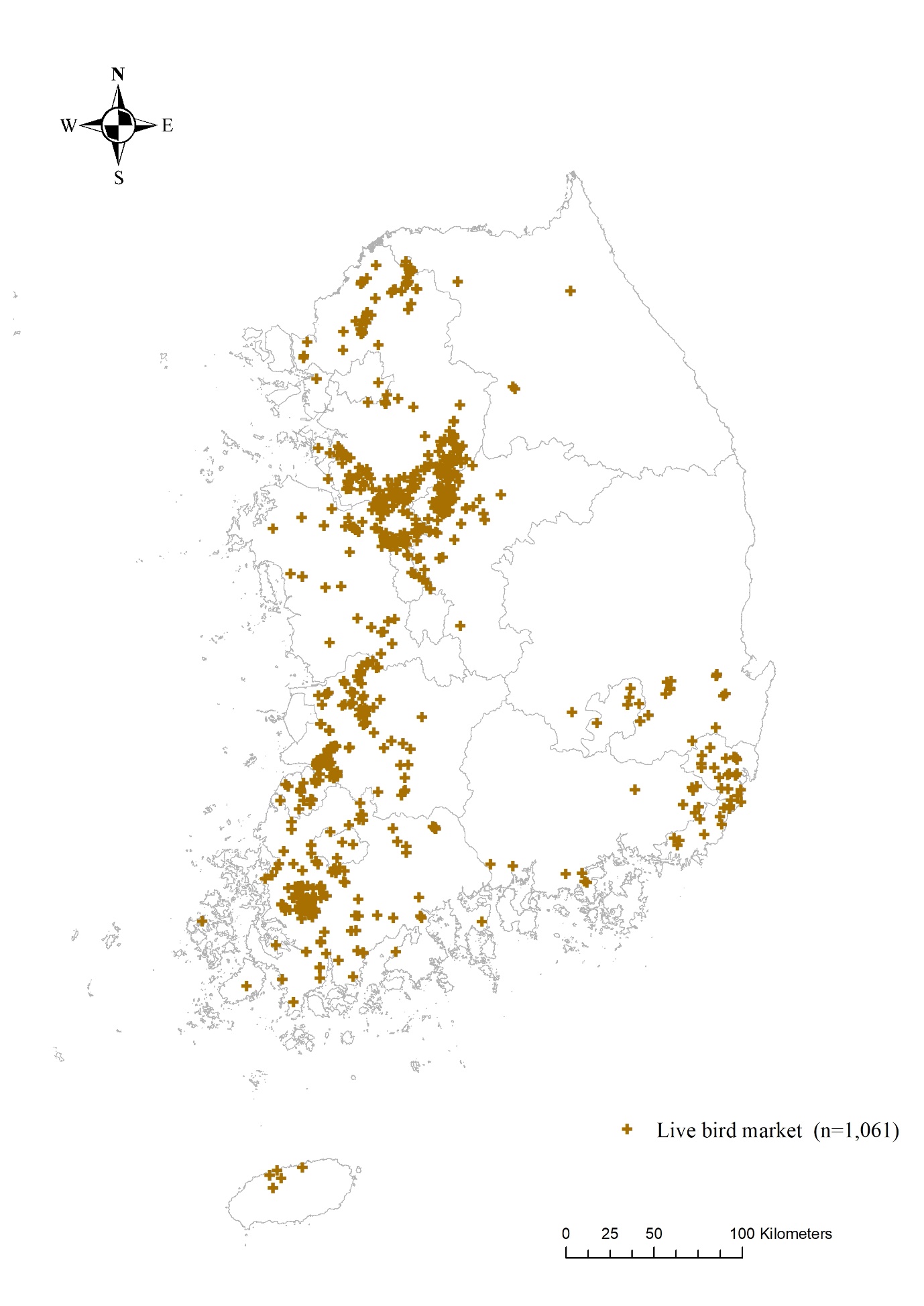


**Figure S12** Live bird markets in South Korea in 2016


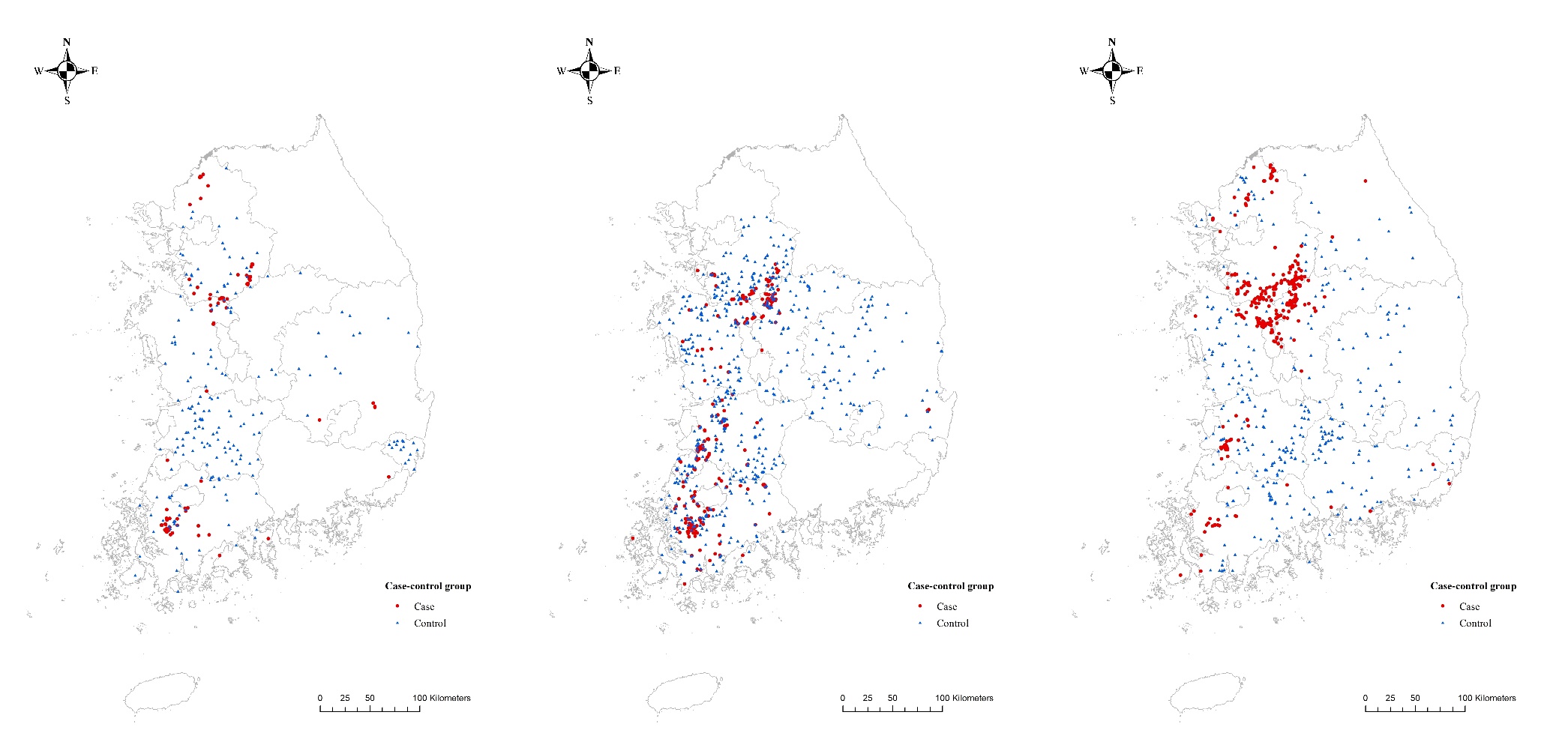


**Figure S13.** Geographical distribution of infected premises and non-infected premises in this study (H5N1 **left** [88 infected premises and 270 non-infected premises], H5N8 **middle** [339 infected premises and 603 non-infected premises], and H5N6 **right** [ 339 infected premises and 339 non-infected premises])

# Results

**Table S2** Univariate logistic analysis for spatial variables in the case-control study of the H5N8 epidemic

| Variable | Odds ratio | | | p-value | VIF |
| --- | --- | --- | --- | --- | --- |
|  | Mean | 95% CI lower | 95% CI upper |  |  |
| Topology |  |  |  |  |  |
| Elevation (m) | 0.991 | 0.988 | 0.993 | < 0.01* | 2.16 |
| Topological wetness index† | 1.376 | 1.262 | 1.505 | < 0.01* | 1.15 |
| Land-use/cover |  |  |  |  |  |
| Proportion of forest body within a 3-km radius (%) | 0.961 | 0.954 | 0.968 | < 0.01* | 2.91 |
| Proportion of rice field within a 3-km radius (%) | 1.045 | 1.034 | 1.056 | < 0.01* | 2.56 |
| Proportion of waterbody within a 3-km radius (%) | 0.999 | 0.944 | 1.054 | 0.98 | - |
| Proportion of wetland within a 3-km radius (%) | 0.983 | 0.888 | 1.078 | 0.73 | - |
| Minimum distance to driveway (km) | 0.845 | 0.662 | 1.069 | 0.17 | 1.14 |
| Poultry and Human |  |  |  |  |  |
| Human (no. of inhabitant/km^2^) | 1.000 | 0.999 | 1.000 | 0.07 | 1.21 |
| Chicken farm (no. of farms/km^2^) | 1.056 | 0.911 | 1.218 | 0.46 | - |
| Domestic duck farm (no. of farms/km^2^) | 12.282 | 7.845 | 19.966 | < 0.01* | 1.27 |
| Wildlife and live bird market |  |  |  |  |  |
| Minimum distance to major wintering site for wild bird (km) | 0.964 | 0.944 | 0.983 | < 0.01* | 1.35 |
| Minimum distance to live bird market (km) | 0.304 | 0.242 | 0.372 | < 0.01* | 1.10 |

Topological wetness index

CI, confidence interval; VIF, variance inflation factor

**Table S3** Univariate logistic analysis for spatial variables in the case-control study of chicken farms during the H5N8 epidemic

| Variable | Odds ratio | | | *P*-value | VIF |
| --- | --- | --- | --- | --- | --- |
|  | Mean | 95% CI lower | 95% CI upper |  |  |
| Topology |  |  |  |  |  |
| Elevation (m) | 0.992 | 0.986 | 0.997 | < 0.01* | 2.18 |
| Heat load index† | 0.176 | 0.010 | 4.709 | 0.235 |  |
| Topological wetness index† | 1.062 | 0.895 | 1.265 | 0.496 |  |
| Land-use/cover |  |  |  |  |  |
| Proportion of forest body within a 3-km radius (%) | 0.972 | 0.957 | 0.985 | < 0.01* | 2.31 |
| Proportion of rice field within a 3-km radius (%) | 1.021 | 1.002 | 1.039 | 0.025 | 1.21 |
| Proportion of waterbody within a 3-km radius (%) | 0.941 | 0.792 | 1.060 | 0.412 |  |
| Proportion of wetland within a 3-km radius (%) | 0.956 | 0.748 | 1.116 | 0.653 |  |
| Minimum distance to driveway (km) | 0.479 | 0.242 | 0.861 | 0.022 |  |
| Poultry and Human |  |  |  |  |  |
| Human (no. of inhabitant/10 km^2^) | 0.995 | 0.926 | 1.035 | 0.856 |  |
| Chicken farm (no. of farms/km^2^) | 1.729 | 1.452 | 2.107 | < 0.01* | 1.47 |
| Domestic duck farm (no. of farms/km^2^) | 9.479 | 2.062 | 46.807 | < 0.01* | 1.10 |
| Wildlife and live bird market |  |  |  |  |  |
| Minimum distance to major wintering site for wild bird (km) | 0.931 | 0.881 | 0.976 | < 0.01* | 1.39 |
| Minimum distance to live bird market (100 m) | 0.748 | 0.669 | 0.818 | < 0.01* | 1.08 |

Topological wetness index

CI, confidence interval; VIF, variance inflation factor

**Table S3.** Univariate logistic analysis for spatial variables in the case-control study of domestic duck farms during the H5N8 epidemic

| Variable | Odds ratio | | | *P*-value | VIF |
| --- | --- | --- | --- | --- | --- |
|  | Mean | 95% CI lower | 95% CI upper |  |  |
| Topology |  |  |  |  |  |
| Elevation (m) | 0.993 | 0.989 | 0.996 | < 0.01* | 1.89 |
| Topological wetness index† | 1.307 | 1.152 | 1.498 | < 0.01* | 1.21 |
| Land-use/cover |  |  |  |  |  |
| Proportion of forest body within a 3-km radius (%) | 0.966 | 0.956 | 0.976 | < 0.01* | 2.93 |
| Proportion of rice field within a 3-km radius (%) | 1.045 | 1.027 | 1.063 | < 0.01* | 2.50 |
| Proportion of waterbody within a 3-km radius (%) | 0.944 | 0.867 | 1.026 | 0.173 | 1.14 |
| Proportion of wetland within a 3-km radius (%) | 1.000 | 0.822 | 1.225 | 0.999 |  |
| Minimum distance to driveway (km) | 0.808 | 0.572 | 1.145 | 0.227 |  |
| Poultry and Human |  |  |  |  |  |
| Human (no. of inhabitant/10 km^2^) | 1.019 | 0.937 | 1.124 | 0.669 |  |
| Chicken farm (no. of farms/km^2^) | 1.438 | 0.812 | 2.700 | 0.233 |  |
| Domestic duck farm (no. of farms/km^2^) | 2.721 | 1.903 | 4.178 | < 0.01* | 1.26 |
| Wildlife and live bird market |  |  |  |  |  |
| Minimum distance to major wintering site for wild bird (km) | 0.985 | 0.955 | 1.016 | 0.327 |  |
| Minimum distance to live bird market (100 m) | 0.927 | 0.908 | 0.944 | < 0.01* | 1.09 |

†Topological wetness index refers to indicators of water accumulation and local drainage.

CI, confidence interval; VIF, variance inflation factor

**Table S4** Summary of marginal posterior distribution of adjusted odds ratio of spatial variables in Bayesian multivariate logistic regression for H5N8 outbreaks

| Chicken farm only  (no. of case= 56, no. of control= 112) | | Duck farm only  (no. of case= 283, no. of control= 514) | | |
| --- | --- | --- | --- | --- |
| Variable | Mean (95% Crls) | | Variable | Mean (95% Crls) |
| Proportion of forest body | 1.005 (0.973, 1.039) | | Topological wetness index | 1.094 (0.939,1.277) |
| Proportion of rice field | 0.976 (0.931, 1.023) | | Proportion of forest | 0.992 (0.972, 1.013) |
| Minimum distance to driveway | 0.657 (0.286, 1.383) | | Proportion of rice field | 0.996 (0.965, 1.028) |
| Chicken farm density | 1.357 (1.084, 1.708) | | Proportion of waterbody | 0.987 (0.870,1.116) |
| Domestic duck farm density | 2.290 (0.710, 9.880) | | Domestic duck farm density | 1.367 (0.993, 1.973) |
| Minimum distance to major wintering site for wild bird | 1.002 (0.921, 1.088) | | Minimum distance to LBM | 0.504 (0.407, 0.607) |
| Minimum distance to LBM | 0.559 (0.544, 0.600) | |  |  |
|  |  | |  |  |
| DIC | 215.09 | | DIC | 402.96 |
| AUC | 0.943 | | AUC | 0.880 |
| Morans`I (p-value) | -0.006 (0.691) | | Morans`I (p-value) | 0.152 (0.491) |


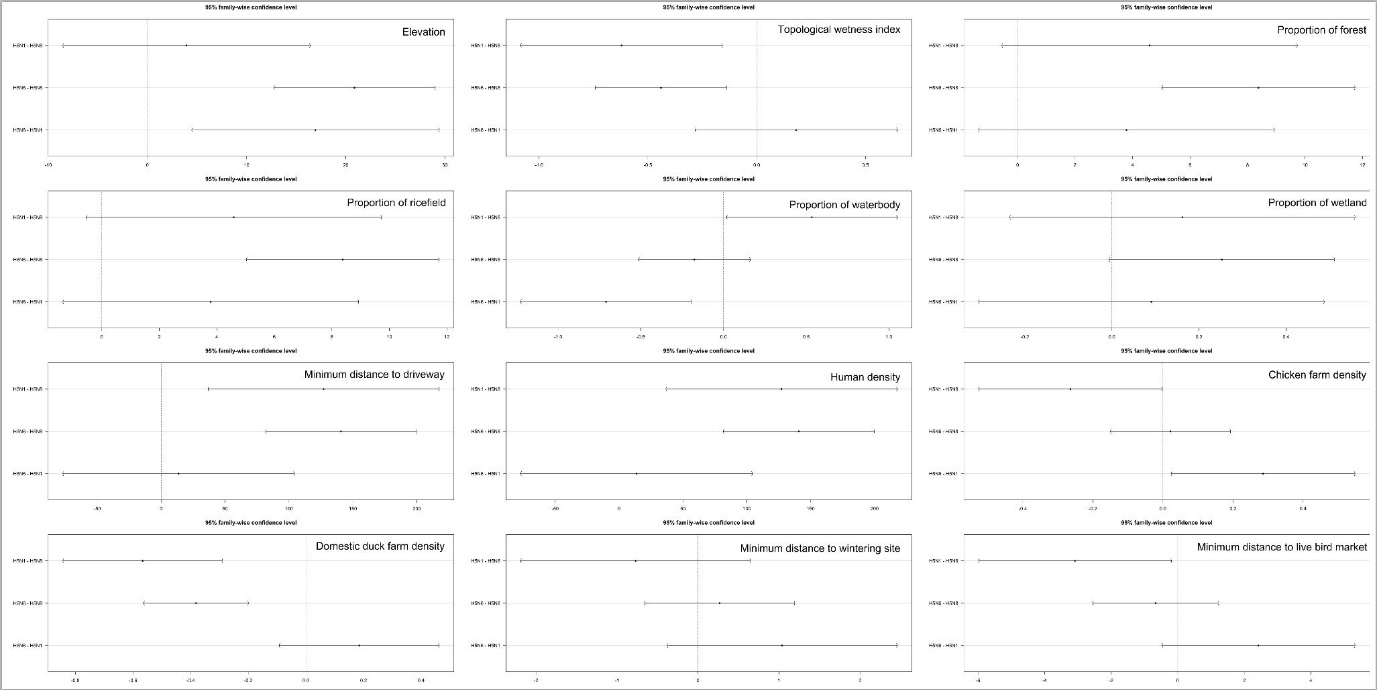


**Figure S14.** Mean difference of spatial variables of infected premises between two paired epidemics using a post-hoc analysis (tukey)


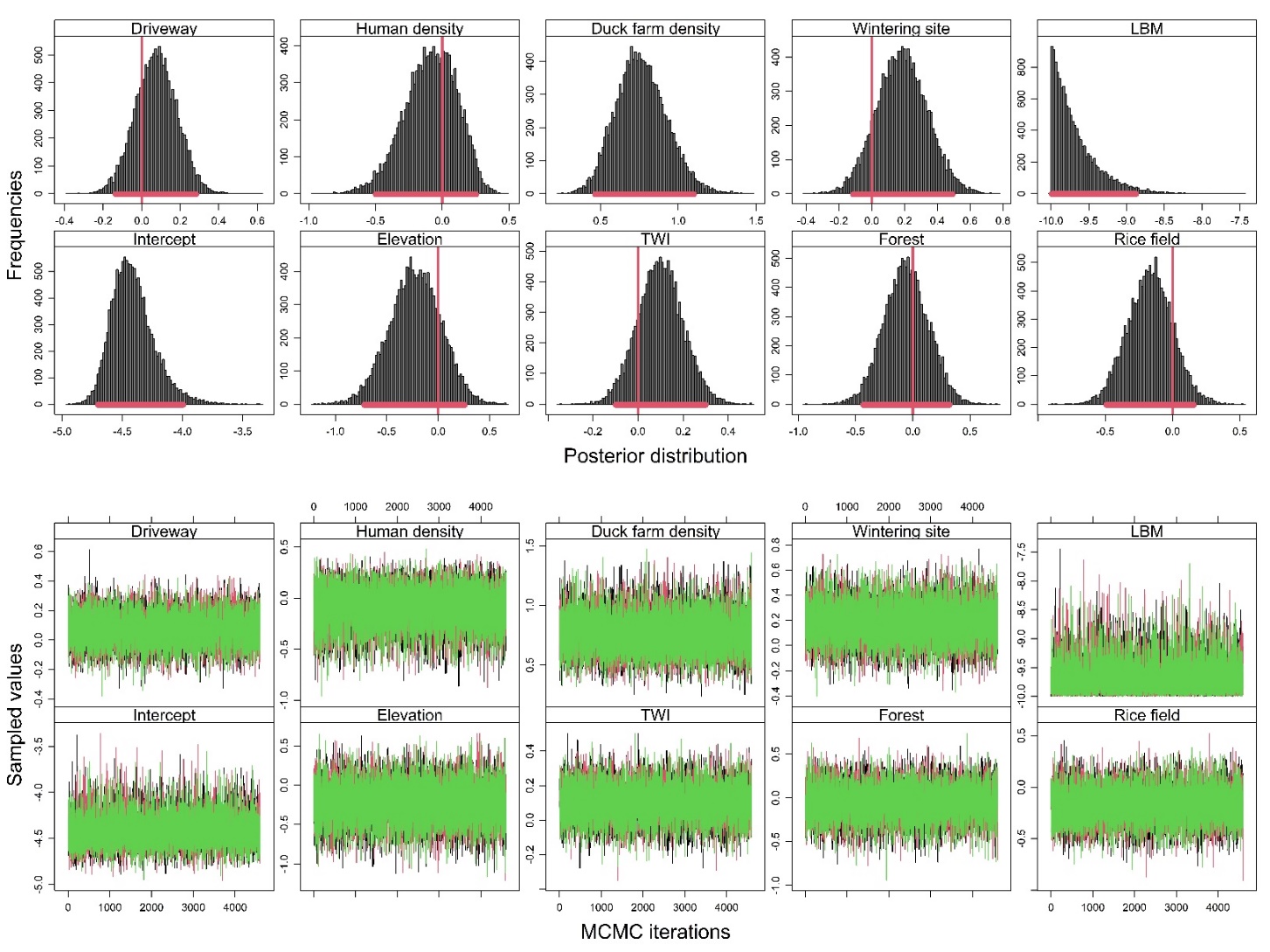


**Figure S15.** Marginal posterior distribution of parameters of spatial variables and MCMC chain


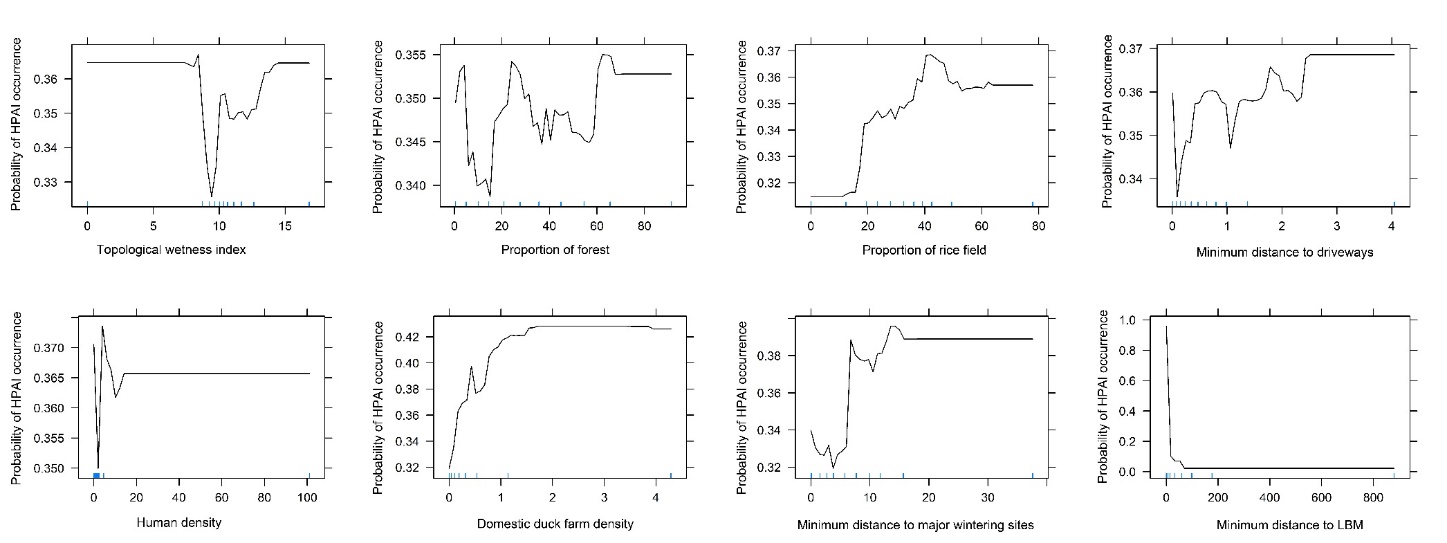


**Figure S16.** Partial dependency plot of spatial variables used in the XGBoost model
